# *In vitro* investigating of anticancer activity of new 7-MEOTA-tacrine heterodimers

**DOI:** 10.1080/14756366.2019.1593159

**Published:** 2019-04-02

**Authors:** Jana Janockova, Jan Korabecny, Jana Plsikova, Katerina Babkova, Eva Konkolova, Dana Kucerova, Jana Vargova, Jan Koval, Rastislav Jendzelovsky, Peter Fedorocko, Jana Kasparkova, Viktor Brabec, Jan Rosocha, Ondrej Soukup, Slavka Hamulakova, Kamil Kuca, Maria Kozurkova

**Affiliations:** a Department of Biochemistry, Institute of Chemistry, Faculty of Science, P. J. Šafárik University, Kosice, Slovak Republic;; b Biomedical Research Center, University Hospital Hradec Kralove, Hradec Kralove, Czech Republic;; c Department of Toxicology and Military Pharmacy, Faculty of Military Health Sciences, University of Defence, Hradec Kralove, Czech Republic;; d Associated Tissue Bank, Faculty of Medicine, P.J. Šafárik University, Kosice, Slovak Republic;; e Department of Cellular Biology, Institute of Biology and Ecology, Faculty of Science, P. J. Šafárik University, Kosice, Slovak Republic;; f Department of Biophysics, Faculty of Science, Palacke University, Olomouc, Czech Republic;; g Department of Organic Chemistry, Institute of Chemistry, Faculty of Science, P. J. Šafárik University, Kosice, Slovak Republic

**Keywords:** 7-MEOTA-tacrine heterodimers, calf thymus DNA, topoisomerases, HL-60, human dermal fibroblasts

## Abstract

A combination of biochemical, biophysical and biological techniques was used to study calf thymus DNA interaction with newly synthesized 7-MEOTA-tacrine thiourea **12**–**17** and urea heterodimers **18**–**22**, and to measure interference with type I and II topoisomerases. Their biological profile was also inspected *in vitro* on the HL-60 cell line using different flow cytometric techniques (cell cycle distribution, detection of mitochondrial membrane potential dissipation, and analysis of metabolic activity/viability). The compounds exhibited a profound inhibitory effect on topoisomerase activity (e.g. compound **22** inhibited type I topoisomerase at 1 µM concentration). The treatment of HL-60 cells with the studied compounds showed inhibition of cell growth especially with hybrids containing thiourea (**14–17**) and urea moieties (**21** and **22**). Moreover, treatment of human dermal fibroblasts with the studied compounds did not indicate significant cytotoxicity. The observed results suggest beneficial selectivity of the heterodimers as potential drugs to target cancer cells.

## Introduction

**Figure 1.**
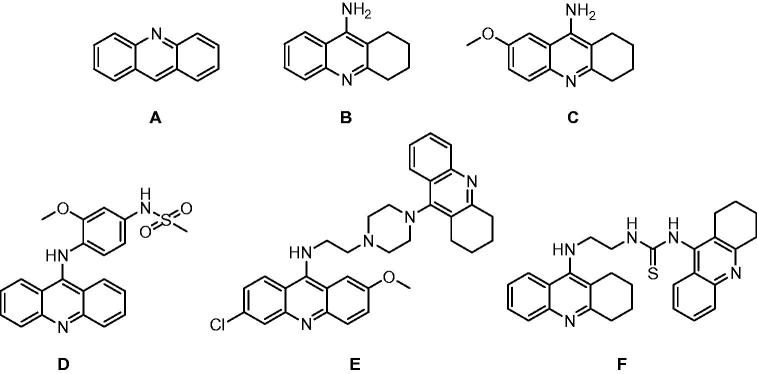
Chemical structures of acridine (A), THA (B) and their derivatives: 7-MEOTA (C)^5^, *m*AMSA (D), 6-chloro-2-methoxy-*N*-{2-[4-(1,2,3,4-tetrahydroacridin-9-yl)piperazin-1-yl]ethyl}acridin-9-ylamine (E)^35^, 1-(1,2,3,4-tetrahydro-acridin-9-yl)-3-[2-(1,2,3,4-tetrahydroacridin-9-ylamino)ethyl]thiourea (F)^35^.

Tacrine (9-amino-1,2,3,4-tetrahydroacridine, THA, Figure 1) was first described as an analeptic able to cause rapid arousal of morphinized dogs and cats^1^^,^^2^. Later, THA was found to be a potent cholinesterase inhibitor of both acetylcholinesterase (AChE, E.C. 3.1.1.7) and butyrylcholinesterase (BuChE, E.C. 3.1.1.8)^3^. Notably, a potential crosstalk between some types of cancer and modulation of AChE activity has been proposed. Accordingly, the inhibition of AChE affecting cholinergic signaling has been associated with some potential benefits in e.g. cancerous lung tissue^4^. The effect of THA has been well-described in neurological diseases^5^. Indeed, THA was licensed in USA and Canada as the first symptomatic treatment for cognitive symptoms associated with Alzheimer’s disease (the drug Cognex)^6^. Clinical use of THA was limited due to its side effects, mainly hepatotoxicity and gastrointestinal symptoms^7^. The precise mechanism of hepatotoxicity still remains unclear^8^. Some researchers associate the THA-associated hepatotoxicity with oxidative bio-activation and the formation of chemically highly reactive metabolites^9^; however, the cell-killing effect is more probably mediated by membrane fluidity alterations^10–12^. Apart from that, mitochondrial dysfunction^13^ and necrosis of liver cells^7^ also emerged as other routes for THA toxicity.

THA is currently used as a versatile scaffold in medicinal chemistry for designing novel hybrid compounds with improved pharmacological and toxicological profiles affecting several pathological mechanisms, e.g. in Alzheimer’s disease pathophysiology^14–18^. The THA derivative 7-MEOTA (7‐methoxy‐1,2,3,4‐tetrahydroacridin‐9‐amine, Figure 1), which was developed in our laboratory and primarily tested to antagonize anticholinergic syndrome evoked by scopolamine, ditrane and 3-quinuclidinyl benzilate^19^^,^^20^ and also as a prophylactic agent against organophosphate poisoning^21^, displayed a better toxicological profile than THA^5^. Mansouri et al.^22^ examined the mechanism of mitochondrial function damage during treatment with THA. Based on the chemical structure, it was postulated that THA might have a similar mechanism of action as the antitumor agent acridine (Figure 1) and related derivatives (e.g. *m*AMSA, imidazoacridinones, *bis*-tacrine, Figure 1)^22^. These acridines are able to intercalate between the planar bases of DNA and to inhibit nuclear type II topoisomerase (Topo II)^23–26^. In addition, a variety of bis-acridines have been developed with anticancer activity^27^^,^^28^. Further research also revealed that THA derivatives might be engaged also in interaction with type I topoisomerase (Topo I)^29^. THA itself was found to be a relatively weak catalytic inhibitor of Topo II implying inhibition of DNA synthesis. The latter led to depletion of mitochondrial DNA and ultimately to apoptosis^22^^,^^29^. Topo I and II are nuclear enzymes which play an important role by formatting superhelical DNA structures and thus ensuring essential cell functions. The topology of DNA can be regulated by certain enzymes leading to mutual transformation of its topological isomers and to the successive relaxation. Catalytic activity of topoisomerases lies in introduction DNA of single (Topo I) or double (Topo II) strand breaks. More recently, topoisomerases emerged as target of great interest in the development of novel antibacterial and anticancer drugs^30^^,^^31^.

Apoptosis is a critical cellular process of programed cell death which features changes in cell morphology such as membrane blebbing, nuclear fragmentation, chromatin condensation and chromosomal DNA fragmentation. Every unwanted regulation of the apoptotic pathway has been associated with either the onset or progression of different diseases including cancer^32–34^. These findings spurred cancer drug discovery in the medicinal chemistry field^35^. However, tetrahydroacridines with their non-planar scaffold can bind to DNA with much lower affinity than acridines^36^.

In this work, new 12 analogs were synthesized by amalgamating THA with the less toxic THA derivative 7-MEOTA via an alkyl chain containing either urea or thiourea moieties. These two parent compounds were formerly anti-Alzheimer’s disease (AD) agents structurally resembling amsacrine or the imidazoacridinones, both being developed as anticancer agents capable to intercalate between DNA-bases and to target Topo II^37^^,^^38^. Our study disclosed their antiproliferative effect against human acute promyelocytic leukemia cell line HL-60 in contrast to their effect on dermal fibroblasts. In summary, herein we provide deep insight into the mode of action of compounds **12**–**22** that might lead to development of novel DNA topoisomerase inhibitors as chemotherapeutic agents.

## Results and discussion

### Chemistry

**Scheme 1.**
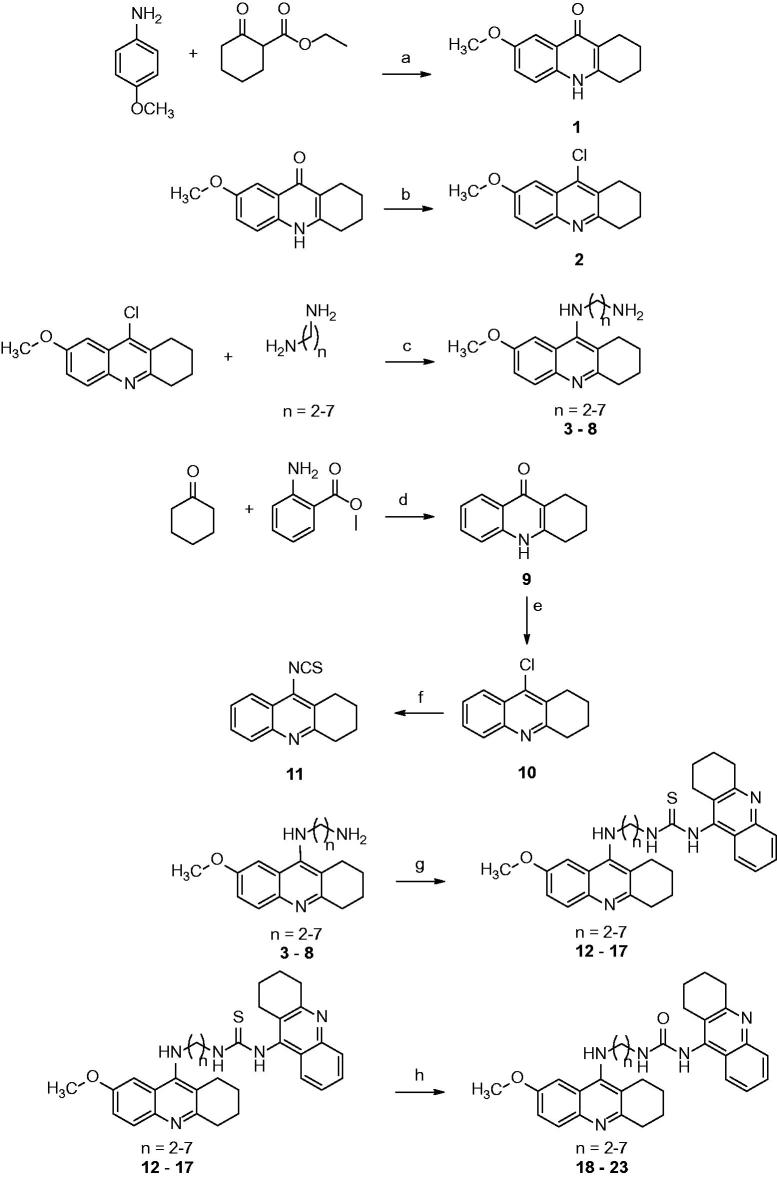
Synthesis of 7-MEOTA-THA thioureas **12**–**17** and ureas **18**–**23**. Conditions: (a) Dean–Stark trap, toluene, reflux 8 h; diphenylether, 220°C 30 min; (b) POCl_3_, reflux 1 h; (c) phenol, 130°C, 2–4 h; (d) P_2_O_5_, *N*,*N*-dimethylcyclohexylamine, 170 °C, 4 h; (e) POCl_3_, reflux 1 h; (f) AgSCN, dry toluene, reflux overnight; (g) 11, CH_2_Cl_2_, RT, 48 h; (h) 2,4,6-trimethylbenzonitrile *N-*oxide, CH_2_Cl_2_, RT, 48 h.

Scheme 1 illustrates the general synthetic procedure for 7-MEOTA-THA hybrids. The starting fused ring of 7-MEOTA, 7-methoxy-1,2,3,4-tetrahydroacridin-10*H*-9-one^1^, was prepared by *p*-toluenesulfonic acid-catalysed condensation reaction of 4-methoxyaniline with ethyl-2-oxocyclohexanecarboxylate in refluxing toluene in good yield (74%). In the next step, **1** was treated with phosphorus oxychloride to give a quantitative yield of 9-chloro-7-methoxy-1,2,3,4-tetrahydroacridine **2**^39^^,^^40^. Treatment of **2** with appropriate 1,ω-diamines in the presence of phenol then provided the desired intermediates *N*-(7-methoxy-1,2,3,4-tetrahydroacridin-9-yl)alkane-1,*ω*-diamines **3**–**8** (71–93%). 1,2,3,4-Tetrahydroacridin-10*H*-9-one **9** was formed directly from the neat reaction of cyclohexanone with *N*-methylanthranilate (65%). Chlorination of **9** was carried out by refluxing with phosphorus oxychloride to obtain 9-chloro-1,2,3,4-tetrahydroacridine **10** in quantitative yield. 9-Isothiocyanato-1,2,3,4-tetrahydroacridine **11** was obtained by refluxing **10** in the dark with silver thiocyanate in anhydrous toluene^41^. The desired 7-MEOTA-THA thioureas **12**–**17** were formed by reaction of the two synthons **11** and diamines **3**–**8** (65–78%). The oxo-analogs were obtained using 2,4,6-trimethylbenzonitrile *N*-oxide to afford 7-MEOTA-THA ureas (**18**–**23**; 88–95%). Structural determination and signal assignments of thiourea hybrids **12**–**17** and urea hybrids **18**–**23** were accomplished by application of the usual combination of ^1^H and ^13^C NMR spectra. Unequivocal assignments were performed by homo- and hetero-correlated two-dimensional NMR experiments (H,H-COSY, H,C-HSQC, H,C-HMBC). The infrared spectrum was obtained only for molecule **11** to observe isothiocyanate group vibrations.

In this work, we have studied DNA binding of novel THA-7-MEOTA dimers **12**–**22**, their influence on the catalytic activity of Topo I/II, their ability to affect cell cycle distribution, and their effect on the mitochondrial membrane potential (MMP) and the metabolic activity and viability of the HL-60 cell line. The results acquired from these *in vitro* studies should enhance understanding of the effects pertinent for drugs targeting cancer. The urea heterodimer **23** was not tested due to its low solubility.

### Biological evaluation

For biological effect analysis of novel compounds **12**–**22**, we prefer to use multiple assays that reflect real physiological/pathological changes in more detail and on single cell level (FACS analysis). It is generally known that most of the chemotherapeutic agents exert their cytotoxic effect either by induction of cancer cell apoptosis or by cell cycle arrest at a specific point^42^. Cumulative evidence has shown that opening of MMP transition pores results in the dissipation of MMP (Δψ_m_) followed by release of pro-apoptotic molecules into the cytoplasm, which leads to programed cell death^43^. To examine whether **12**–**22** affect mitochondrial physiological processes, HL-60 cells were treated with the studied compounds for 24, 48 and 72 h and labeled with TMRE. In general, mitochondria with normal MMP retain the dye, resulting in strong fluorescence. Compounds inducing a collapse in MMP allow flow of the dye from mitochondria to cytoplasm leading to depolarization of the mitochondrial membrane and fluorescence decrease. The results from MMP analysis clearly show that **14**–**17** and **21** at 15 µM and **22** at 5 µM were able to evoke MMP dissipation in more than approximately 60% of cells after 24 h treatment and caused cell death with an efficiency between 80 and 100% at 15 µM for urea analogs and 25 µM for thioureas after 24, 48 and 72 h (Figure 2(A)). Their effect is comparable to that recorded for the acridine which have been explored as a potential therapeutic agent for the treatment of cancer^44^ (Table 1). The other tested heterodimers **12**, **13**, **18**–**20** and parent THA induced a negligible effect on MMP and a weak cytotoxic effect (data not shown). A simultaneous analysis of viability (staining with PI) and the activity of cellular metabolism showed a very similar trend to acquired changes in MMP analysis identifying the effectivity of compounds **14**–**17** and **21**, **22** to kill cancer cells (Figure 2(B)). IC_50_ from both techniques over different time periods are displayed in Table 1. The cytotoxic effects on HL-60 cells go hand-in-hand with the lipophilicity of the individual compounds within both subseries, the most toxic being **17** and **22** from the urea and thiourea families, respectively. The most pronounced cytotoxic effect was associated with tether lengthening, with urea derivatives **21** (at 15 µM) and **22** (at 5 µM) having five and six methylenes in the linker being found to exert the most cytotoxic profile. The higher cytotoxic effect presumably reflects easier cell membrane penetration. Interestingly, the cytotoxic effect against HL-60 also correlated well with the ability to inhibit activity of topoisomerases. It is known that inhibitors of Topo I, such as camptothecin, exhibit cell cycle arrest induced apoptosis^45^. Cryptolepine, a plant-derived alkaloid, has recently been considered as a promising anticancer agent due to a demonstrated effect of Topo II, inhibition of DNA-synthesis, inhibitory effects on the cell cycle, and potent toxicity to a number of cancer cells^46^. Hu^47^ demonstrated that some bis-tacrine derivatives exhibited moderate selectivity toward colon and melanoma cells and interestingly, butyl-linked bis-tacrine showed strong cytotoxicity against non-small cell lung, colon cancers and melanoma. A variety of dimeric compounds have been shown a broad spectrum activity against different human tumor cells [e.g. bis(phenazine-1-carboxamides) act as dual Topo I/II-directed anticancer drugs and were evaluated for growth inhibitory activity in cancer cell lines including P388 leukemia and Lewis lung carcinoma^48^] and also dimeric naphthalimides^49^ and bis-acridines have been recognized for clinical trials as anticancer candidates^47^.

Figure 2.Effect of 7-MEOTA-THA thioureas **14**–**17** (5–25 μM), ureas **20**–**22** (1–15 μM) and THA (5–10 μM) on changes in A: MMP and B: viability in HL-60 cells. Cells were analyzed 24, 48 and 72 h after treatment with studied compounds. The results were calculated as mean ± SD from three independent experiments. Statistical significance *p* < .05 (*), .01 (**), and .001 (***) for the particular experimental group compared to untreated control (C).

**Figure:**
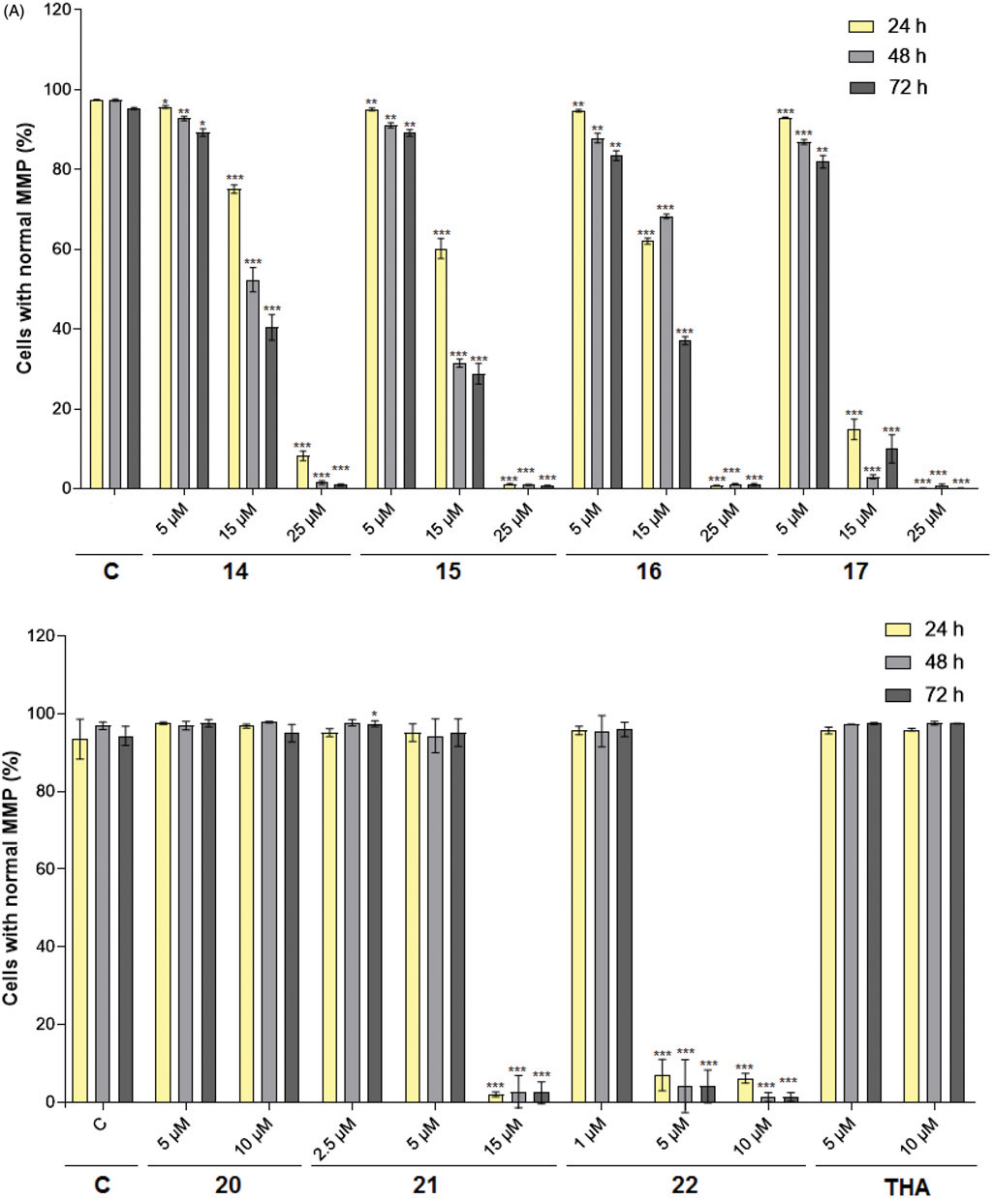

**Figure:**
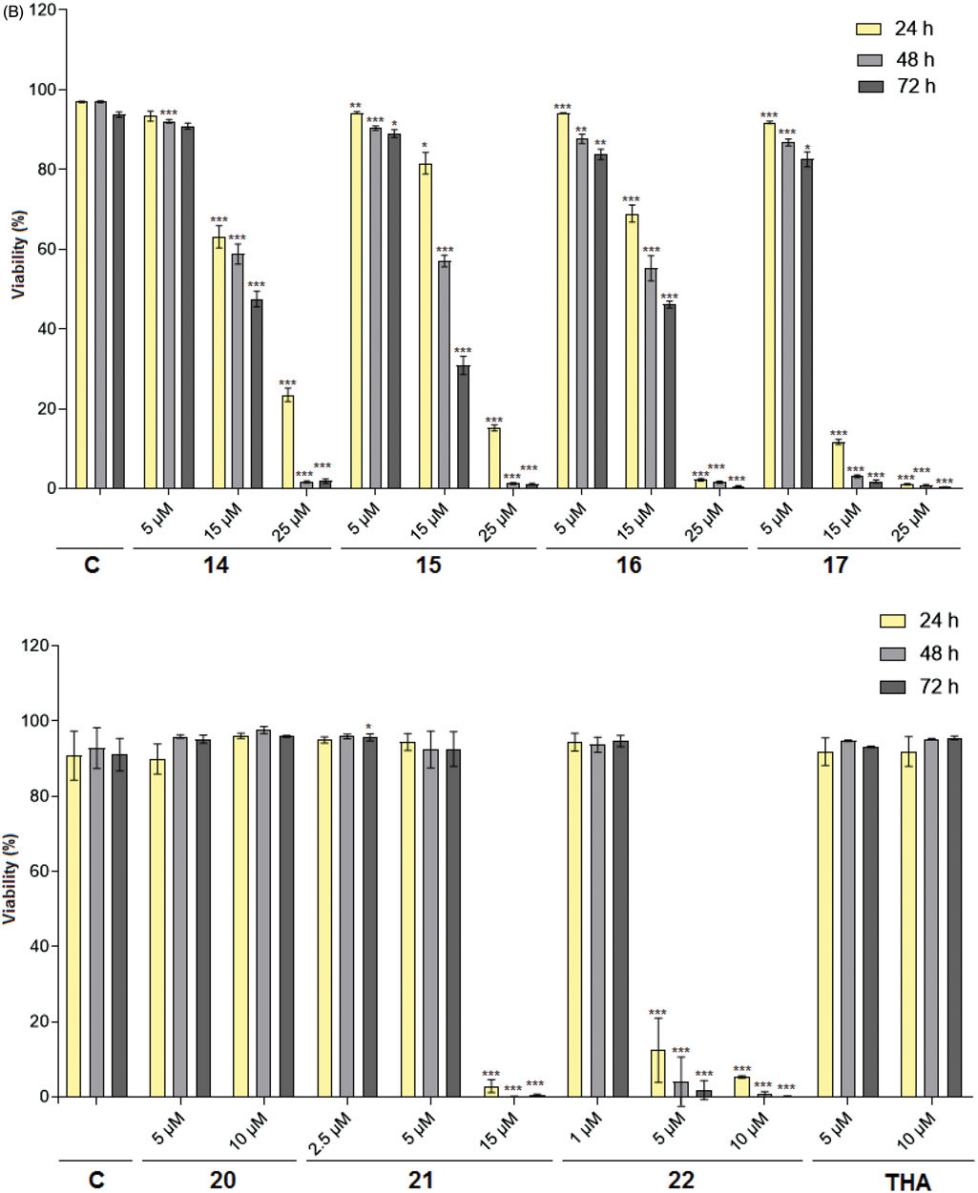

**Table 1.** IC_50_ values for MMP dissipation (TMRE) and viability (FDA/PI) of HL-60 cells after 24, 48 and 72 h incubation with studied 7-MEOTA-tacrine thio-/ureas **12**–**22**.

Compound	Time (h)	IC_50_ (μM)±SEM	
		TMRE	FDA/PI
**12**	24	47.29 ± 2.15	43.94 ± 1.04
	48	30.46 ± 4.78	33.39 ± 1.02
	72	38.00 ± 4.68	47.82 ± 2.83
**13**	24	43.95 ± 1.98	40.98 ± 0.95
	48	29.90 ± 3.95	30.16 ± 3.41
	72	26.97 ± 6.37	29.75 ± 1.49
**14**	24	17.89 ± 3.04	18.11 ± 6.61
	48	15.48 ± 4.13	15.87 ± 3.75
	72	14.56 ± 5.04	15.12 ± 3.74
**15**	24	15.81 ± 3.47	19.51 ± 4.33
	48	13.54 ± 2.08	15.74 ± 2.49
	72	13.33 ± 4.13	13.57 ± 4.01
**16**	24	15.88 ± 1.23	16.64 ± 3.01
	48	16.54 ± 2.16	15.71 ± 5.31
	72	14.33 ± 2.94	15.12 ± 2.69
**17**	24	10.18 ± 3.22	9.58 ± 1.27
	48	7.59 ± 1.62	7.64 ± 1.69
	72	7.36 ± 2.82	7.24 ± 2.71
**21**	24	9.19 ± 5.39	9.80 ± 4.13
	48	7.11 ± 5.47	6.87 ± 3.04
	72	9.18 ± 2.75	6.85 ± 1.92
**22**	24	3.95 ± 2.23	4.19 ± 2.64
	48	3.76 ± 1.17	3.78 ± 2.74
	72	3.94 ± 2.79	3.67 ± 2.40
Tacrine	24	n.d	n.d
	48	n.d	n.d
	72	n.d	n.d
Acridine	24	n.d.	n.d.
	48	6.37 ± 2.51	6.40 ± 2.09
	72	5.23 ± 3.18	5.04 ± 4.96

n.d.: not defined; TMRE: tetramethylrhodamine ethyl ester perchlorate; FDA: fluorescein diacetate; PI: propidium iodide; **23** was not tested due to its low solubility.

The cytotoxicity of **12**–**22** was also determined on cultured human dermal fibroblasts by measuring MMP. As shown in Supplementary Figure S1, all the studied compounds were less toxic to non-cancer cells compared to cancer cells at given concentrations after 72 h treatment, which underlies their advantage in development of anticancer drugs.

In order to address the effect of compounds **12**–**22** to impair cell proliferation, flow cytometry analysis of the cell cycle distribution was hence used. Based on the results from MMP dissipation and viability analyzes, HL-60 cells were treated with different concentrations of **12**–**17** (5–25 µM) and **18**–**22** (1–15 µM) for 24, 48 and 72 h. After 48 h incubation, results for urea derivatives **21** (15 µM) and **22** (10 µM) clearly demonstrated a significant accumulation of cells in G1 phase (Supplementary Figure S2(B)). In comparison, thiourea analogs **14**–**17** revealed a concentration effect (Supplementary Figure S2(A)). These compounds significantly altered cell cycle progression, inducing block of the cell cycle in S phase after just 24 h treatment at 25 µM concentration. THA and acridine were used as controls. Despite the relatively high concentration of THA, it did not exert any effect in this experiment (48 h incubations with THA is shown Supplementary Figure S2(B)).

**Figure 3.**
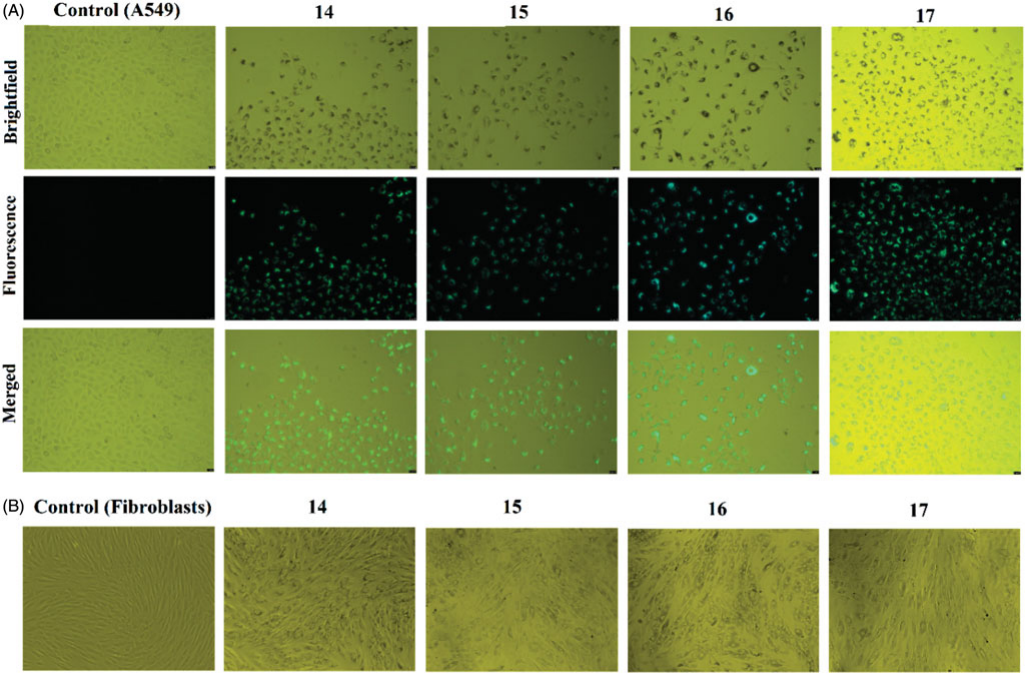
Intracellular accumulation after 24 h incubation of **14**–**17** in A549 cells (5 µM, A) visualized by fluorescence microscopy, and in fibroblasts (25 µM, B) visualized by phase contrast microscopy. Images were obtained by the fluorescence microscope Leica DMI6000B (HCX PL APO CS 10.0 × 0.40 DRY UV objective) and Leica DMI3000B. Presented images were obtained from three independent experiments.

**Figure 4.**
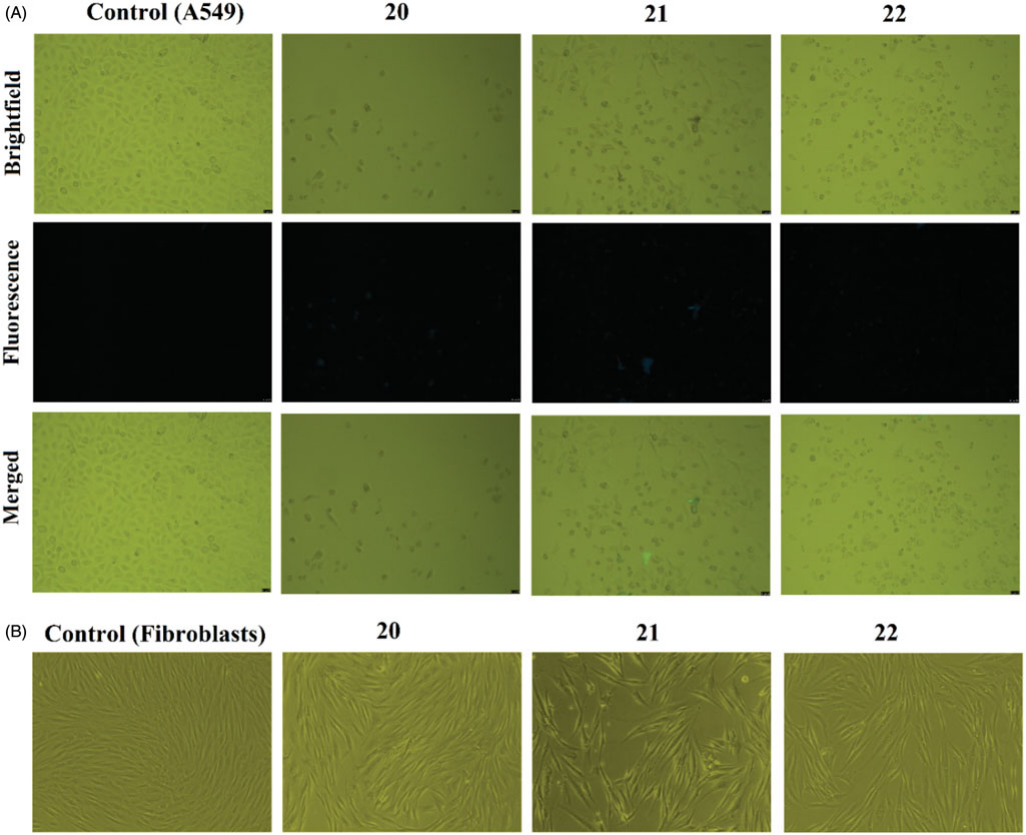
Intracellular accumulation after 24 h incubation of **20**–**22** in A549 cells (5 µM, A) visualized by fluorescence microscopy, and in fibroblasts (5 µM, B) visualized by phase contrast microscopy. Images were obtained by the fluorescence microscope Leica DMI6000B (HCX PL APO CS 10.0 × 0.40 DRY UV objective) and Leica DMI3000B. Presented images were obtained from three independent experiments.

The intracellular distribution of **12**–**22** was analyzed on the model of A549 adherent cancer cells and human dermal fibroblasts by a simple approach. After 24 h incubation with the derivatives, the cells were washed in order to dispose of unbound compounds, so that only compounds that were able to permeate into the cells could be visualized. The fluorescence of each sample was examined in each channel but images were captured using only two channels: (1) blue excitation range [BP excitation filter 450–490 nm (cube I3) and LP suppression filter 515 nm]—when heterodimers were present in the samples they exhibited positivity only in this channel; (0) brightfield mode was also applied in order to capture the cells themselves, visualizing single cells regardless of fluorescence of the studied compounds. According to the signal in channel 1 (I3 filter cube), compounds **14**–**17** (5 µM) showed abundant accumulation after 24 h incubation and a strong fluorescence signal in the A549 cells. All these heterodimers were visible as dark spots even in brightfield mode (Figure 3(A)). Phase contrast microscopy was used to visualize the intracellular distribution of **12**–**22** in human dermal fibroblasts as well. Thiourea hybrids **14**–**17** displayed accumulation also in fibroblasts at higher concentrations (25 µM, Figure 3(B)), whereas in the case of A549 cells, such concentrations were lethal. Hybrids **12**, **13**, **18** and **19** exhibited only selective accumulation in A549 cells. Interestingly, heterodimers **12** and **13** accumulated in fibroblast cytoplasm (similarly to **14**–**17**) whereas urea heterodimers **18**, **19** did not (data not shown). It was also observed that ureas **20**–**22** accumulated neither in A549 cells nor in fibroblasts (Figure 4). Taking all these facts together, it was noticed that the thiourea hybrids with longer chains permeate through the cell membrane more easily than their urea counterparts. This phenomenon might be sufficiently explained by the higher lipophilicity of thiourea hybrids compared to urea derivatives, as has been experimentally determined in many studies^50^. Interestingly, changes in morphology were observed in both cancer and non-cancer cells treated with **14**–**17**. In the case of A549, treatment resulted in a more rounded shape of the cells, suggesting that the hybrids impaired cell viability. These changes in morphology confirmed our previous results in which the same concentration of derivatives decreased the viability and MMP of A549 cells after 24 h of incubation. The size of human dermal fibroblasts was increased after permeation of compounds **14**–**17** into the cells. In general, this led to a loss of typical fibroblast-like morphology. However, penetration of the derivatives into fibroblasts did not affect MMP, in contrast to A549 cells. These findings cannot be sufficiently explained due to the unclear mode of action at the intracellular level. Moreover, hybrids **14**–**17** seem to be good candidates for further studies of the exact localization of these compounds inside the cells, using confocal microscopy and other techniques.

### Activity of Topo I and II

Given that the studied 7-MEOTA-THA thio-/urea hybrids **12**–**22** are able to interact with ctDNA (see section below), it is important to consider whether they are also able to inhibit some of the cell nucleus enzymes involved in DNA operations, and which alter its topology through decatenation and relaxation of supercoiled DNA, as for example Topo I/II. Moreover, Topo I/II are also employed in other cell functions, such as replication, transcription, recombination and chromosomal segregation^51–53^. In recent years, Topo I/II have been under increasing study as a cell target for anticancer drugs capable of targeting their catalytic cycle. Some clinical studies showed that such inhibition ability could be associated with the cytotoxicity effect of inhibitors^54^. Compounds inhibiting the catalytic functions of Topo I/II can cause permanent breaks in DNA structure which can lead to cell death, but the detailed mechanism remains unknown^55–57^. Topo I/II inhibitors are classified as catalytic inhibitors (they decrease enzyme activity) or poisons (they stabilize the DNA-cleavage complex and protect repeated DNA religation)^58^.

#### Effect on Topo I relaxation activity

**Figure 5.**
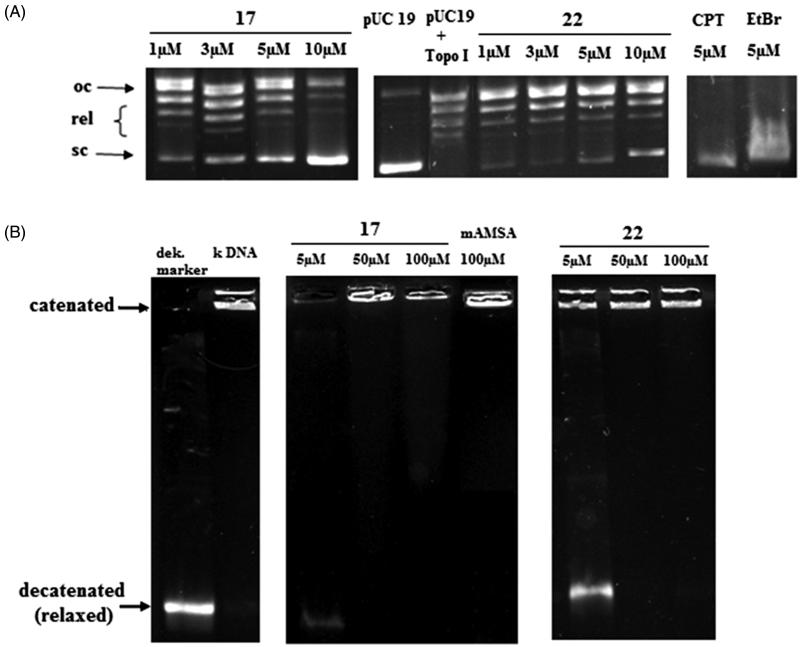
Effect of **17** and **22** on A: relaxation of supercoiled plasmid DNA by calf thymus Topo I. Supercoiled plasmid DNA pUC19 (lane pUC19) was incubated with calf thymus Topo I in the absence (lane pUC19 + Topo I) and in the presence of various compound concentrations (0–10 μM); B: decatenation of *k*DNA (0.16 μg) by human Topo IIα. Catenated kinetoplast DNA (lane *k*DNA) was incubated with human Topo IIα in the absence (lane *k*DNA + Topo II) and in the presence of various compounds concentration (0–100 μM).

In this study, we examined the effect of 7-MEOTA-THA thio-/urea derivatives **12**–**22** on the catalytic activity of Topo I by measuring the Topo I-mediated relaxation of supercoiled plasmid pUC19. Due to significant and dose-dependent inhibition of Topo I catalytic activity all the compounds under study reduced the amount of relaxed DNA, while simultaneously increasing the amount of supercoiled DNA. 7-MEOTA-THA thioureas **12**–**17** inhibited the catalytic activity of Topo I at 60 µM (Supplementary Figure S3). Compounds **14**–**17** were able to inhibit the catalytic relaxation ability of Topo I on pUC19 already at 5 µM, and hence further testing was carried out at concentrations of 1–10 µM (Supplementary Figure S4). The most effective from this subset was compound **17** due to strong inhibition at 1 µM (Figure 5(A)). On the other hand, the urea analogs **18**–**22** showed strong inhibition potency at 30 µM except for **20** which inhibited Topo I partially at that concentration (Supplementary Figure S3). Intriguingly, compound **22** was effective at 1 µM concentration (Figure 5(A)). The well-established Topo I inhibitor camptothecin and DNA intercalator EtBr, both applied at 5 µM were used as positive controls. Results were compared with standards THA (Topo I inhibition at 60 µM, not shown) and 7-MEOTA (Topo I inhibition at 30 µM, not shown)^59^.

#### Effect on Topo II decatenation activity and Topo II drug screening

It is well-established that catalytic Topo II inhibitors interfere with the binding between Topo II and DNA (e.g. suramin, aclarubicin), inhibit ATP binding (e.g. novobiocin), or stabilize the noncovalent DNA-Topo II complex (e.g. dexrazoxane, merbarone). Topo II poisons are able to stabilize the covalent DNA-Topo II cleavage complex and are clinically used for their antitumor activity (e.g. etoposide, teniposide, doxorubicin)^60^.

The ability of **12**–**22** to affect the decatenation of catenated kinetoplast DNA (*k*DNA) from the insect trypanosome *Crithidia fasciculata* by Topo II was examined (Figure 5 and Supplementary Figure S5). ATP-dependent decatenation assay is a specific assay for detection of compounds with potential Topo II inhibition activity. Topo II-targeted compounds are able to interact with (at least) one step of the topoisomerase catalytic cycle. The decatenation of *k*DNA by Topo II generates different products (nicked, relaxed or supercoiled form of DNA) which move easily into the agarose gel compared with the larger-sized catenated *k*DNA^61^. Interestingly, slight evidence of decatenated products was observed in the presence of the studied compounds **12**–**22** at 5 µM compared to 200 µM etoposide, 100 µM ellipticine and 100 µM mAMSA (Topo II poisons/inhibitors used as standards). All the compounds revealed the same pattern of inhibition of catalytic activity of Topo II at 50 and 100 µM concentrations. Vispe et al.^62^ studied a series of novel bis- and tetra-acridine anticancer drugs, which were found not only as DNA interacting agents but also as Topo II-mediated DNA decatenation inhibitors. These acridines inhibited Topo II activity at 32 µM, which is comparable with our obtained results. Given our findings, we propose that Topo I and II both represent an important target for our studied heterodimers.

Topo II poisons (such as etoposide or *m*AMSA) are able to stabilize the DNA-Topo II complex and thus form linear DNA due to the simultaneous cleavage of both strands of double-stranded DNA. Plasmid DNA pHOT1 (the DNA substrate) used in the cleavage assay contains a single high affinity Topo II cleavage site^63^. In our study, the linear form of DNA was found when 100 µM of etoposide or 100 µM of mAMSA was applied, but not for **12**–**22** (Supplementary Figure S6). These results indicated that **12**–**22** are characterized with Topo II catalytic inhibition, but not as Topo II poisons. Topo II poisons were found to be important for activating secondary leukemias involving certain chromosomal translocations. On the other hand, Topo II catalytic inhibitors are able to change the cytotoxic effect of poisons and overcome multidrug resistance^60^^,^^64^^,^^65^. Numerous Topo II catalytic inhibitors are nowadays used in combination with chemotherapeutic agents (e.g. aclarubicin, suramin, MST-16)^66–68^.

### Spectroscopic properties

**Figure 6.**
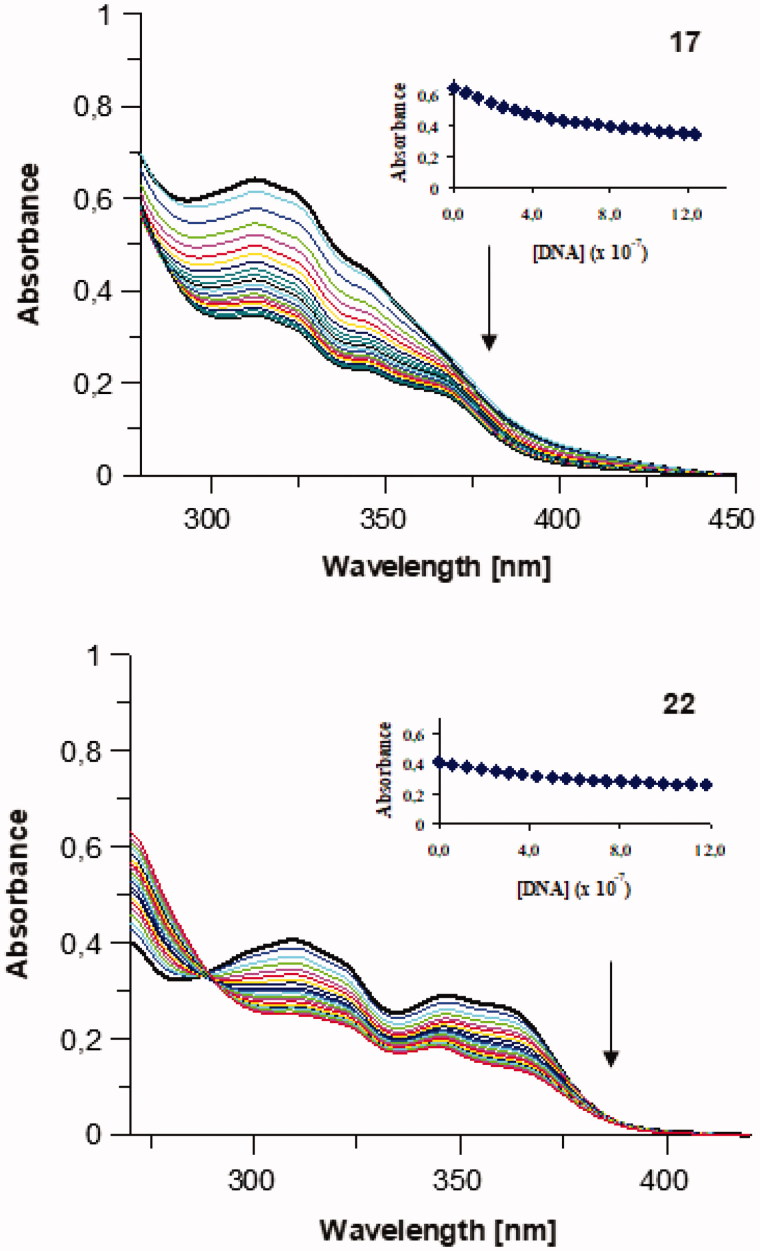
UV–vis absorption titration spectra of studied 7-MEOTA-THA thiourea **17** (49 µM) and urea **22** (49 µM) in 10 mM Tris–HCl buffer (pH 7.4; RT) with increasing concentration of ctDNA (0–1.24 µM). Arrows indicate the changes after addition of increasing concentration of ctDNA. Inserted graphs represent the non-linear fitting of absorption titration, the plot of [DNA] versus appropriate absorbance.

Several binding modes have been identified between native and/or synthetic double-stranded DNA (dsDNA) and low molecular-weight compounds acting as chemotherapeutic agents or gene regulators. The non-covalent DNA interactions are represented mainly by intercalation between the base pairs of dsDNA; and binding to minor/major grooves of dsDNA, stabilized by a mix of electrostatic, hydrophobic, hydrogen-bonding interactions or outside random binding^69^. The interactions of 7-MEOTA-THA thio-/ureas **12**–**22** with ctDNA were studied in order to investigate their possible binding modes. Representative UV–vis absorption titration spectra for thiourea hybrid **17** and urea analog **22** are displayed in Figure 6. The addition of increasing amounts of ctDNA to a constant concentration of the compounds (UV–vis spectroscopic titration) revealed a hypochromic shift ranging from 16.31 to 46.10%. A mild bathochromic shift to higher wavelengths was observed only for compounds **17**, **18**, **20** and **21** (Δλ = 1 nm). The observed changes suggested that studied compounds **12**–**22** are able to interact with ctDNA, although from the small bathochromic shifts we can rather point out to groove binding or electrostatic interaction, rather than intercalation. The DNA-binding characteristics of the studied 7-MEOTA-THA thio-/ureas **12**–**22** are summarized in Table 2. The binding constants ***K*** of the studied compounds in complex with ctDNA ranged between 0.5 and 8.0 × 10^6^ M^−1^ and were comparable with the measured ***K*** of acridine and greater than the literature-cited ***K*** for acridin-3,6-diyl dithiourea hydrochlorides (2.9–7.6 × 10^5^ M^−1^)^70^, proflavine diureas (0.9–4.2 × 10^5^ M^−1^)^71^ and tacrine derivatives with ctDNA (0.16–4.0 × 10^5^ M^−1^)^29^. In addition, it is shown that linking of THA and 7-MEOTA with thio-/urea moieties positively influenced the interaction power. Binding constants typical for DNA intercalators lie in the range 10^4^–10^6^ M^−1^ and they are usually lower than binding constants typical for groove binders (range from 10^5^ to 10^9^ M^−1^).

**Table 2.** DNA-binding characteristics of 7-MEOTA-THA thioureas **12**–**17** and ureas **18**–**22**.

Compound^a^	λ_max_		Bathochromic shift (nm)	Hypochromicity (%)	*K* (M^−1^)
	Free	Bound			
**12**	312	312	0	16.31	3.8 × 10^6^
**13**	312	312	0	21.83	5.5 × 10^6^
**14**	312	312	0	32.40	6.5 × 10^6^
**15**	312	312	0	33.52	8.0 × 10^6^
**16**	312	312	0	38.99	4.0 × 10^6^
**17**	313	312	1	46.10	4.3 × 10^6^
**18**	311	312	1	31.33	4.5 × 10^6^
**19**	311	311	0	30.10	0.5 × 10^6^
**20**	310	311	1	30.19	0.6 × 10^6^
**21**	311	312	1	29.06	3.0 × 10^6^
**22**	309	309	0	38.06	6.0 × 10^6^
**THA**	324	329	5	40.25	3.8 × 10^4^
**Acridine**	400	407	7	50.02	4.5 × 10^6^
**7-MEOTA**	334	336	2	13.10	7.9 × 10^4^

a The concentrations of studied compounds were as follows: compounds **12**–**17**: 49 µM; **18**–**20**: 99 µM; **21**, **22**: 49 µM; **23** was not tested due to its low solubility, THA and acridine: 50 µM; 7-MEOTA: 25 µM.

DsDNA tertiary structure is stabilized via hydrogen bonds and base stacking interactions. When a solution of dsDNA is exposed to extreme heat, the double helix dissociates to the single-stranded form (known as denaturated DNA). This leads to disruption of intermolecular forces and hydrogen bonding interactions between the DNA base pairs^72^. The stability of the dsDNA structure is determined by the melting temperature (*T_m_*). DNA denaturation is usually measured spectrophotometrically at the temperature-dependent excitation point of 260 nm. In this work, *T_m_* of ctDNA was found to be 68 °C whereas in the presence of 7-MEOTA-THA thioureas **12**–**16** slightly increased (Δ*T_m_* was 1–2 °C). In the presence of 7-MEOTA-THA ureas **18**–**22**, no increase of *T_m_* of ctDNA was observed, except for compound **22** which exhibited a profound increase of *T_m_* of more than 10 °C. Thermal characterization of the studied compounds **12**–**22** and the first derivative of ctDNA denaturation curves in the presence of the studied compounds are shown in Supplementary Figure S7. Usually, ligands which could bind to DNA through intercalation significantly increase *T_m_* of DNA, and Δ *T_m_* is typically around 5–8 °C, due to stabilization of dsDNA. Groove binding and electrostatic binding ligands produce mostly negligible effects on *T_m_* of DNA^72^. Tao et al.^73^ revealed that a subtle decrease of *T_m_* (0.9 °C) in melting temperature analysis with ctDNA for resmethrin characterizes this compound as a groove binder. Results from our study indicated that only compound **22** could possibly bind strongly to ctDNA in intercalative mode, but it is not excluded that 7-MEOTA thio-/ureas **12**–**21** are able to insert between the planar bases of ctDNA.

**Figure 7.**
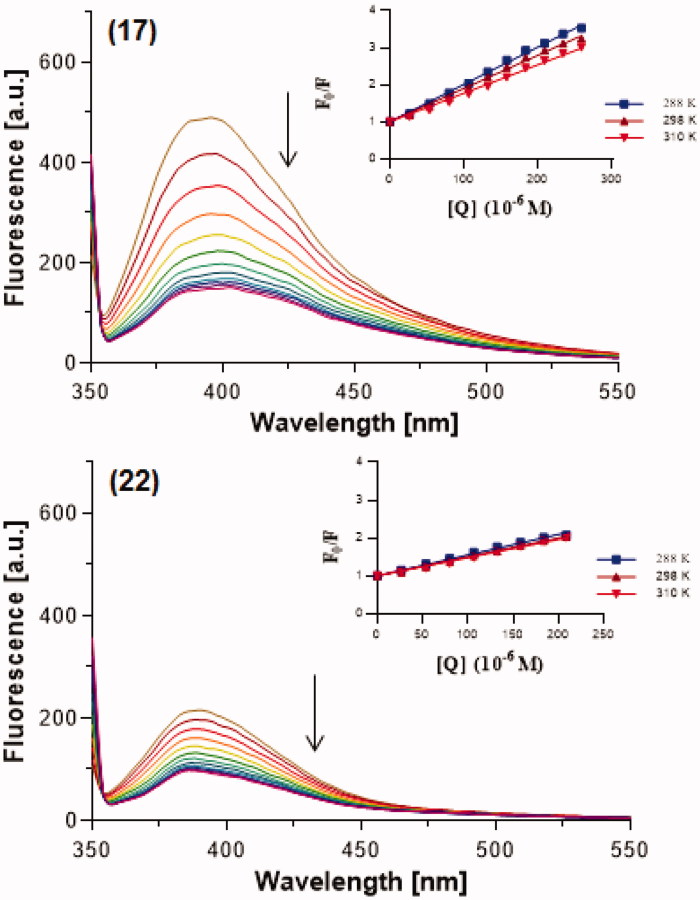
Steady-state fluorescence spectra of studied 7-MEOTA-THA thiourea **17** and urea **22** (5 µM, upper lines) in 10 mM Tris–HCl buffer (pH 7.4) with increasing concentration of ctDNA (0–280 µM, color lines). Arrows show the changes in emission intensity upon increasing ctDNA concentration. The inserted graphs represent the Stern–Volmer plot (plot F_0_/F versus [Q]) at three different temperatures (288, 298 and 310K).

A more sensitive evaluation of the interaction of studied 7-MEOTA-THA thio-/ureas **12**–**22** with ctDNA was accomplished using steady-state fluorescence measurements. Fluorescence spectra of studied heterodimers at fixed concentration with increasing concentration of ctDNA showed a progressive quenching in the fluorescence intensity which was directly proportional to the ctDNA concentration. Representative results are displayed in Figure 7 (for hybrids **17** and **22**) and suggest interaction between the compounds and ctDNA. Data from these fluorescence quenching experiments were also used for determination of ***K_SV_***that provides a measure of the quenching ability of ctDNA. The inserted graphs in Figure 7 present the Stern–Volmer plots obtained as a function of F_0_/F versus [Q]. These plots are characterized by an intercept of one on the *y*-axis and the slopes are equal to ***K_SV_***, as summarized in Table 3.

**Table 3.** Stern–Volmer (*K_SV_*) and quenching constants (*k_q_*) of complex ctDNA: 7-MEOTA-THA thio-/ureas **12**–**22** at three different temperatures and pH 7.4.

Compound*	Temperature (K)	*K_SV_*(×10^3^ M^−1^)	*k_q_*(×10^11^ M^−1^ s^−1^)	*R*^2^
**12**	288	5.58	5.58	0.991
	298	4.83	4.83	0.989
	310	3.77	3.77	0.994
**13**	288	7.18	7.18	0.986
	298	6.69	6.69	0.995
	310	5.66	5.66	0.995
**14**	288	10.70	10.70	0.999
	298	8.92	8.92	0.996
	310	7.81	7.81	0.992
**15**	288	7.47	7.47	0.999
	298	7.33	7.33	0.995
	310	6.16	6.16	0.993
**16**	288	8.75	8.75	0.997
	298	7.09	7.09	0.998
	310	5.97	5.97	0.998
**17**	288	10.08	10.08	0.997
	298	8.98	8.98	0.994
	310	7.69	7.69	0.993
**18**	288	6.72	6.72	0.992
	298	6.18	6.18	0.995
	310	5.26	5.26	0.993
**19**	288	9.54	9.54	0.993
	298	8.76	8.76	0.993
	310	7.34	7.34	0.987
**20**	288	5.31	5.31	0.991
	298	5.26	5.26	0.998
	310	4.77	4.77	0.993
**21**	288	8.05	8.05	0.997
	298	7.30	7.30	0.997
	310	6.56	6.56	0.996
**22**	288	5.48	5.48	0.993
	298	5.00	5.00	0.998
	310	4.81	4.81	0.991

* The concentration of studied compounds **12**–**22** was 5 µM, **23** was not tested due to its low solubility, *R*^2^: correlation coefficient.

Fluorescence quenching is observed in two processes, as dynamic or static quenching. Static quenching is based on the fact that the fluorophore and added quencher form a stable non-fluorescent complex in the ground state which promptly goes back to the ground state without photon emission after light absorption. For dynamic (collisional) quenching, quencher diffusion to the fluorophore is typical during the lifetime of an excited state and the rate of this quenching is dependent upon temperature, viscosity and diffusion. The decrease of fluorescence in the case of dynamic quenching is due to random collision between the fluorophore and quencher^74^. A Stern–Volmer plot can depend on the mechanism of quenching, and can be non-linear or linear, and upward or downward curving. In this work, we observed linear Stern–Volmer plots indicating that only one type of quenching process is possible, either dynamic or static quenching. Both static and dynamic quenching can be recognized with different temperatures. Higher temperature of solution leads to a higher diffusion coefficient, and an increase of ***K_SV_*** (by increasing the temperature random collision is more probable). On the other hand, increased temperature goes hand in hand with decreased complex stability and thus the static quenching constant is lower^75^^,^^76^. With increasing temperature, from the obtained data showing decreasing ***K_SV_*** values it is clear that ctDNA can quench fluorescence of the studied compounds as a static quencher. To further confirm the quenching process ***k_q_*** was calculated; this indicates the accessibility of a fluorophore to a quencher, or the efficiency of quenching. Calculated ***k_q_*** are listed in Table 3 and were found to be 3.77–10.70 × 10^11^ M^−1^ s^−1^ at different temperatures. The limiting diffusion rate constant of biomolecules in the case of dynamic quenching was found to be 2.00 × 10^10^ M^−1^ s^−1^. However, there is no limit for static quenching^77^^,^^78^. Since the obtained values of ***k_q_*** were higher than the limiting diffusion rate constant, it is suggested that the quenching process in this study was static rather than dynamic.

The modified Stern–Volmer equation gives the relationship between fluorescence emission intensity and the concentration of quencher and was used to determine ***K_b_*** of the forming complex between 7-MEOTA-THA thio-/urea hybrids and ctDNA. The calculated ***K_b_*** and **n** are recorded in Table 4. The nature of the forces useful for the formation of the complex between the studied compounds and ctDNA can be identified by the thermodynamic parameters, **ΔH**, **ΔS** and **ΔG**. Values of all thermodynamic parameters are summarized in Table 5. The negative values of **ΔG** are characteristic of a favorable and spontaneous binding reaction. Positive values of both **ΔS** and **ΔH** are typical for hydrophobic interaction, while negative values of both can be regarded as indicating van der Waals forces and hydrogen bonds. However, positive values of **ΔH** and negative values of **ΔS** describe electrostatic interaction between ionic species in an aqueous solution^79^^,^^80^. Hence, the obtained results indicate that binding between the studied 7-MEOTA-THA thio-/ureas **12**–**22** and ctDNA was spontaneous due to negative **ΔG** values at all three temperatures, and the positive values of **ΔH** and **ΔS** showed that hydrophobic interactions play a central role in the binding of the studied 7-MEOTA-THA thio-/ureas **12**–**22** with ctDNA. Based on the negative **ΔH** and **ΔS** for **16** and **20**, we can assume that interaction in these ligand-ctDNA complexes is mediated by van der Waals forces (Table 5).

**Table 4.** Binding constants and various thermodynamic parameters for studied 7-MEOTA-THA heterodimers **12**–**22** in complex with DNA at three different temperatures.

Compound*	Temperature (K)	*K_b_*(×10^4^ M^−1^)	*n*	*R*^2^
**12**	288	0.36	0.95	0.995
	298	0.48	1.00	0.993
	310	0.69	1.07	0.996
**13**	288	1.13	1.05	0.989
	298	1.39	1.08	0.997
	310	2.16	1.15	0.999
**14**	288	0.77	0.96	0.999
	298	2.34	1.11	0.999
	310	3.46	1.17	1.000
**15**	288	1.32	1.07	0.999
	298	2.11	1.12	0.997
	310	2.70	1.17	0.999
**16**	288	2.61	1.13	0.998
	298	1.83	1.11	0.998
	310	1.06	1.07	0.999
**17**	288	2.12	1.09	0.998
	298	3.02	1.14	0.997
	310	4.02	1.19	0.999
**18**	288	1.90	1.12	0.997
	298	2.31	1.15	0.997
	310	2.51	1.18	0.997
**19**	288	2.31	1.10	0.997
	298	4.02	1.18	0.997
	310	5.92	1.24	0.999
**20**	288	0.69	1.03	0.995
	298	0.49	0.99	0.999
	310	0.13	0.83	0.996
**21**	288	1.65	1.08	0.996
	298	1.83	1.10	1.000
	310	2.03	1.13	0.999
**22**	288	0.49	0.99	0.997
	298	1.59	1.13	0.997
	310	2.45	1.19	0.999

* The concentration of studied compounds **12**–**22** was 5 µM, **23** was not tested due to its low solubility, *R*^2^: correlation coefficient.

**Table 5.** Various thermodynamic parameters for studied 7-MEOTA-THA heterodimers **12**–**22** in complex with DNA at three different temperatures.

Compound*	Temperature (K)	ΔH (kJ mol^−1^)	ΔS (J K^−1^ mol^−1^)	ΔG (kJ mol^−1^)
**12**	288	22.64	146.75	−64.85
	298			−66.31
	310			−68.07
**13**	288	22.11	154.01	−66.49
	298			−68.03
	310			−69.87
**14**	288	50.49	250.77	−122.75
	298			−125.26
	310			−128.27
**15**	288	23.98	162.50	−70.80
	298			−72.43
	310			−74.38
**16**	288	−30.59	−21.39	36.76
	298			36.97
	310			37.23
**17**	288	21.56	157.81	−67.03
	298			−68.61
	310			−70.50
**18**	288	9.43	114.84	−42.52
	298			−43.67
	310			−45.05
**19**	288	31.71	193.90	−87.59
	298			−89.52
	310			−91.85
**20**	288	−57.61	−125.09	93.65
	298			94.91
	310			96.41
**21**	288	6.88	104.68	−37.05
	298			−38.10
	310			−39.35
**22**	288	54.07	259.46	−128.83
	298			−131.43
	310			−134.54

* The concentration of studied compounds **12**–**22** was 5 µM, **23** was not tested due to its low solubility.

**Figure 8.**
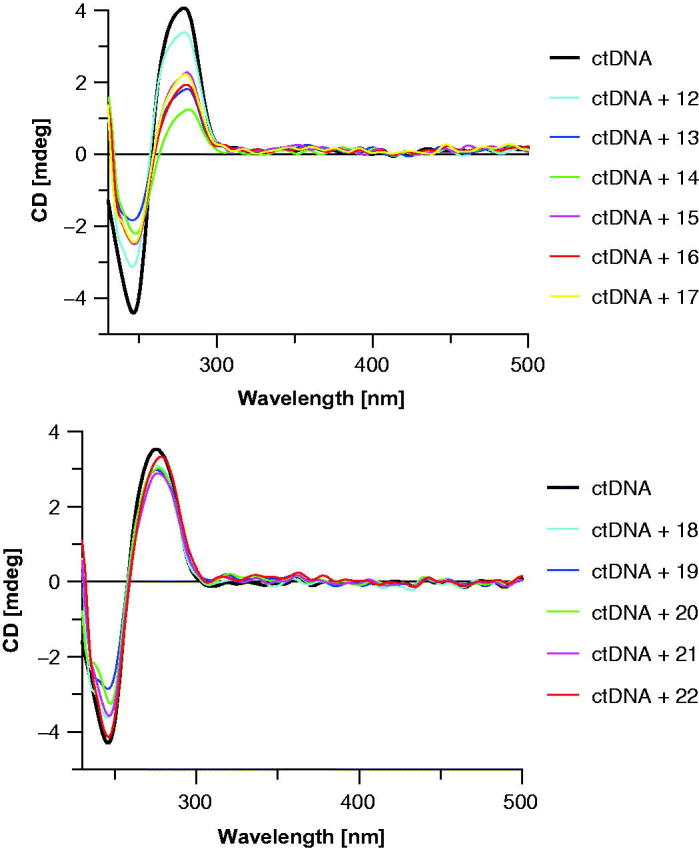
CD spectrum of ctDNA (black line, 7.46 µM) in the absence and in the presence of studied 7-MEOTA-THA heterodimers **12**–**22** (color lines, 0.3 mM) in 10 mM Tris–HCl buffer (pH 7.4).

Polarized light spectroscopy is a suitable technique for fast characterization of nucleic acids and their complexes with small molecules. From linear dichroism (LD) can be deduced structure information about the relative conformation between the DNA axis and the ligand. Circular dichroism (CD) is another powerful tool to gain insight into the structural determinants characterizing such complexes. Whereas LD can be used to monitor the angles between DNA-base transition states relative to the axis orientation which are identical with the helix axis in flow or electric field-oriented DNA, the induced CD of the same transition could provide information about molecular orientation relative to the surrounding nucleobases^81^. CD is the difference in the absorption of left-handed circularly polarized light (L-CPL) and right-handed circularly polarized light (R-CPL): CD = A_L-CPL—_A_R-CPL_^82^. CD spectra of ctDNA were modified by addition of 7-MEOTA-THA thio-/ureas **12**–**22** to study the orientation mode and overall conformation changes produced due to the influence of conjugates. Representative CD spectra for hybrids **12**–**22** can be found in Figure 8. The studied derivatives are achiral and thus do not exhibit any CD signal of their own. The B-DNA conformation is characteristic with the CD spectrum in the region 220–300 nm. The positive band of ctDNA at approximately 275 nm is caused by base stacking, whereas the negative band around 245 nm is indicative of the helicity of the B-DNA^83^. Incubation of **12**–**17** with ctDNA resulted in CD spectral shifts in both positive and negative CD bands. The positive band of ctDNA at 280 nm showed a visible decrease of molar ellipticity and the negative band at 246 nm exhibited an apparent increase of molar ellipticity after modulation with thioureas **12**–**17**. Kozurkova et al.^71^ reported previously that proflavine compounds in complex with ctDNA resulted in changes in both CD bands, and in our obtained results the decrease of peak ellipticity at 280 nm may be explained as disruption of stacked bases due to possible intercalation of thiourea analogs to ctDNA in order to optimize the binding interaction. Accordingly, the changes of peak ellipticity at 246 nm came about due to hydration binding of DNA which affects its helicity. When modulated by urea derivatives **18**–**22** under the same conditions, no significant changes in the subset were recorded within ctDNA, which indicates that the nature of binding of the urea hybrids to ctDNA is non-intercalative. However, these small visible changes at both bands may be attributed to certain conformational changes.

**Figure 9.**
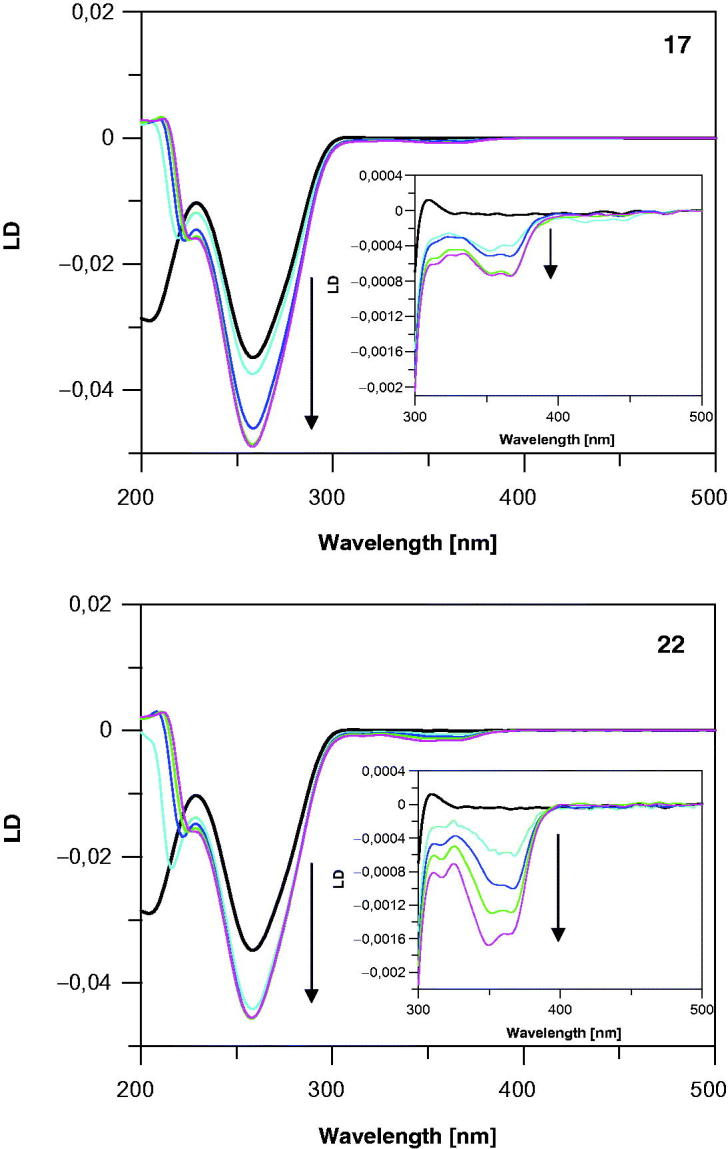
LD spectrum of ctDNA (black line, 310 µM) in the absence and in the presence of studied 7-MEOTA-THA thio-/ureas **17** and **22** (color lines, 0–0.1 mM) in 10 mM Tris–HCl buffer (pH 7.4).

LD is defined as LD = A_//_−A_⊥_, where A_//_and A_⊥_ are absorbance values measured with the light plane polarized parallel and perpendicular, respectively, to the oriented ctDNA in this study. The orientation of measured ctDNA in the absence and in the presence of the studied 7-MEOTA-THA thio-/ureas **12**–**22** was obtained by flow using a cuvette cell. Representative spectra are shown in Figure 9. The appearances of the LD spectra for all complexes of ctDNA with the studied compounds were almost identical, characterized by a negative signal in both the DNA absorption region (220–300 nm) and in the Soret region (320–450 nm). The existence of LD signals in these regions proves that **12**–**22** interact with DNA and become oriented after anchoring to DNA. The negative values of these LD signals are consistent with perpendicular orientations of the chromophoric molecules **12**–**22** relative to the DNA helix axis as expected for intercalative binding mode^84^. Moreover, the LD signal of DNA (at 258 nm) increases in magnitude upon addition of each of the compounds **12**–**22** indicating either lengthening and/or an increase in rigidity of double-helical DNA, which can usually be related to unwinding due to intercalation^85^^,^^86^. The observed changes upon addition of the heterodimers to DNA were not clearly correlated with their chemical structures. However, neither the perpendicular orientation of transition moments of the compounds to the helix axis nor increased rigidity of the DNA helix represents an unequivocal proof for DNA intercalation^84^. Therefore, the data are interpreted also with respect to the results of other experiments.

## Experimental

### Materials

All chemicals and reagents purchased were of reagent grade and used without further purification. The applied material and cell lines are listed in Supplementary data (see “Experimental, Materials” section).

### Methods

#### DNA and tested sample solutions

CtDNA was dissolved in TE buffer (Tris–EDTA) overnight at 4 °C to completely solubilize. The concentration of ctDNA was determined from its absorbance at 260 nm using extinction coefficient ε = 6600 M^−1^ cm^−1^. The purity of ctDNA was identified by monitoring the value A_260_/A_280_. Stock solutions of tested 7-MEOTA-THA thio-/ureas **12**–**22** were prepared in DMSO (stock concentration of samples was 30 mM). Further dilutions were prepared in the appropriate aqueous buffer.

#### Flow cytometry analysis

For the flow cytometry analysis, HL-60 cells were seeded in six-well plates (TPP, Trasadingen, Switzerland) at 1 × 10^6^ per well and changes in the total cell number, metabolic activity, MMP, cell cycle distribution and cell death were analyzed at 24, 48 and 72 h after treatment with the studied 7-MEOTA-THA thio-/ureas **12**–**22**, as described previously^87^^,^^88^. Human dermal fibroblasts (5 × 10^4^) were seeded into a 12-well plate (Sarstedt, Deutschland) and treated with the studied 7-MEOTA-THA thio-/ureas **12**–**22** for 72 h, and changes in MMP were analyzed.

*Immunophenotype characterization of human fibroblasts*. Isolated dermal fibroblasts were characterized using flow cytometry. After dissociation with trypsin–EDTA (Gibco, Waltham, MA), the cells were washed twice with PBS (Gibco, Life Technologies, Carlsbad, CA) supplemented with 2% (v/v) FBS. Aliquots of 2.0 × 10^5^ cells were incubated with mouse anti-human CD90/FITC, CD105/PE, CD73/APC, CD26/PE, and with a cocktail of hematopoietic markers: CD14-/CD20-/CD34-/CD45/PerCP (Miltenyi Biotec GmbH, Bergisch Gladbach, Germany) for 10 min, washed with 1–2 mL of PBS and centrifuged at 3000*g* for 10 min. The resuspended cell pellet was analyzed in a Becton Dickinson FACSCalibur using CellQuest software (Becton Dickinson).

*Cell cycle distribution*. HL-60 cells were treated with various concentrations of the studied seven MEOTA-THA thio-/ureas (**12**–**17**: 5–25 µM; **18**–**22**: 1–15 µM) for 24, 48 and 72 h. Untreated cells were also included in this test for comparison. The method used to perform the experiment has been published previously^89^. ModFit 3.0 (Verity Software House, Topsham, ME) software was used to generate DNA content frequency histograms and to quantify the number of cells in the individual cell cycle phases.

*Analysis of MMP (ΔΨ_m_).* For detection of ΔΨ_m_, HL-60 cells/fibroblasts were treated with various concentrations of the studied 7-MEOTA-THA thio-/ureas (HL-60: **12**–**17**: 5–25 µM, **18**–**22**: 1–15 µM; fibroblasts: **12**–**17**: 15 and 25 µM, **18**–**22**: 2.5 and 5 µM) for 24, 48 and 72 h (for fibroblasts only 72 h). The method used to perform the experiment has been published according to a previously described protocol^87^^,^^89^.

*Analysis of metabolic activity and viability*. Analysis of metabolic activity and viability of HL-60 cells was performed using fluorescein diacetate (FDA; BD Pharmingen, San Diego, CA) and PI double-staining. HL-60 cells were treated with various concentrations of the studied 7-MEOTA-THA thio-/ureas (**12**–**17**: 5–25 µM; **18**–**22**: 1–15 µM) for 24, 48 and 72 h and subsequently harvested by centrifugation, washed with HBSS and stained with FDA (100 ng mL^−1^) in HBSS buffer for 20 min in darkness at RT. Prior to measurements, cells were stained with PI (1 µg cm^−3^) and analyzed using a BD FACSCalibur flow cytometer. Fluorescence of FDA was detected via a 530/30 nm band pass filter (FL-1) and PI via a 670 nm long-pass filter (FL-3). Results were analyzed using FlowJo software (TreeStar Inc.).

#### Statistical analysis

Results were presented as the mean ± SD (standard deviation) of at least three independent experiments. Statistical significance was determined by Student’s *t*-test and results were considered significant if *p*<.05*, *p*<.01** and *p*<.001***.

#### Intracellular accumulation

A549 cells (6 × 10^3^ per well) were seeded onto microscopy glass slides with mounted 12-well silicone chamber (Ibidi GmbH, Planegg, Germany) and left to settle for 24 h. They were then treated with the studied 7-MEOTA-THA thio-/ureas **12**–**22** (5 µM) or with the medium alone (control group) for 24 h and washed with pre-warmed PBS in order to remove the unbound fraction. Intracellular accumulation of studied heterodimers in A549 cells was evaluated by fluorescence microscope Leica DMI6000B (Leica Microsystems GmbH, Wetzlar, Germany) using two channels as stated in Supplementary Table S1. Every sample was captured using the same settings for HCX PL APO CS 10.0 × 0.40 DRY UV objective. Results were analyzed using Leica Application Suite Lite (LAS AF Lite) software. Each presented image represents a single sample and consists of separated images from a channel and their mutual overlap.

Fibroblasts (4 × 10^4^ per well) were seeded into a 12-well plate, left to settle for 24 h and treated with the studied 7-MEOTA-THA thio-/ureas **12**–**22** (**12**–**17**: 15 and 25 µM, **18**–**22**: 2.5 and 5 µM) or with the medium alone (control group). After 24 h of incubation with the studied derivatives, the cells were analyzed with an inverted fluorescence microscope Leica DMi3000B (Leica Microsystems).

#### Topoisomerase I-mediated supercoiled pUC19 relaxation

The influence of the studied 7-MEOTA-THA thio-/urea hybrids on the catalytic activity of Topo I was identified according to a previously described protocol^29^^,^^90^. Reaction mixtures were incubated for 45 min at 37 °C in the absence or in the presence of the studied 7-MEOTA-THA thio-/urea derivatives **12**–**22** (1–60 µM). The gel was photographed under UV and gel images were obtained using a photogel documentation system (Syngene, Cambridge, UK).

#### Influence of derivatives on topoisomerase II decatenation

Freshly prepared 5× complete assay buffer for Topo II decatenation containing 0.16 µg catenated kinetoplast DNA (kDNA) and 2 U of human Topo IIα in the absence or presence of the studied 7-MEOTA-THA thio-/ureas (**12**–**22**: 5, 50 and 100 µM) was incubated for 45 min at 37 °C. The method used to perform the experiment has been published previously^29^^,^^90^.

#### Topoisomerase II-mediated DNA cleavage assay

DNA cleavage reactions were carried out in a 20 µL final volume containing freshly prepared 5× complete assay buffer, 0.2 µg supercoiled pHOT-1 and 5U human Topo IIα. Reactions were incubated in the absence or in the presence of the studied 7-MEOTA-THA thio-/ureas (**12**–**22**: 10 and 100 µM) at 37 °C for 30 min. The method used to perform the experiment has been published previously^29^^,^^90^.

#### UV–vis absorption spectroscopy and determination of binding constants

UV–vis spectroscopic analyses were realized in 10 mM Tris–HCl buffer (pH 7.4; RT). UV–vis absorption titration spectra were measured on a Varian Cary 100 spectrophotometer in a 100-QS quartz cuvette with optic length 1 cm. UV–Vis spectra of newly developed 7-MEOTA-THA thioureas (**12**–**17**: 49 µM) and ureas (**18**–**20**: 99 µM; **21**, **22**: 49 µM) in the absence or in the presence of an increasing concentration of ctDNA (0–1.24 µM) were recorded in the wavelength range 230–450 nm.

The absorbance A measured at any wavelength indicates both the free (Af) and DNA-bound (A_b_) types:

(1) $$A=A_{f}+A_{b}=\varepsilon_{f}.{\;C}_{f}+\varepsilon_{b}.{\;C}_{b}$$

where C is the constant compound concentration (C = C_f_+C_b_) and ε_f_ and ε_b_ are the appropriate extinction coefficients (Equation (1)). For determination of C_f_ and r was used the equation (Equation (2)):

(2) $$\alpha =\frac{C_{b}}{C}=\frac{1-C_{f}}{C}=\frac{A_{f}-A}{A_{f}-A_{b}}$$

where α is the fraction of binding compound, and A_f_ and A_b_ are the absorptions for free and fully bound compound at the checking wavelength. Subsequently, r = αC/C_DNA_ and C_f_ = (1–α)C, where C_DNA_ is the total DNA concentration^91^. Data from spectroscopic titration were used for determination of binding constants ***K*** of the derivative-ctDNA complexes using a Scatchard plot in the form r/C_f_ (where r is the number of ligand molecules which bind to one mol of nucleotide as a function of added titrant, and C_f_ is the molar concentration of free ligand), the McGhee and von Hippel equation (Equation (3)^92^, and then using a fitting function incorporated into graphic software GnuOctave 2.1.73:

(3) $$\frac{r}{C_{f}}=K(1-\mathrm{nr}){\left[\frac{1-\mathrm{nr}}{1-\left(n-1\right)r}\right]}^{n-1}$$

In this equation, n is the number of binding sites for binding of one ligand molecule to DNA, r is the molar ratio of the bound ligand to the polynucleotide and C_f_ is the molar concentration of free ligand.

#### T_m_ measurements

DNA melting temperature measurements were performed in a quartz cuvette (1 cm path length) on a Varian Cary 100 spectrophotometer equipped with a heating multiple cell block apparatus. Measurements were realized in BPE buffer (pH 7.1; 1 mM EDTA, 2 mM NaH_2_PO_4_, 6 mM Na_2_HPO_4_). The temperature was increased at a rate of 1 °C min^−1^ over the range 40–90 °C. The absorbance was measured at 260 nm for ctDNA alone (concentration of ctDNA in measurements with **12**–**17**: 93.2 µM and with **18**–**22**: 0.32 µM) and for ctDNA in complex with the studied derivatives (concentration of **12**–**17**: 37.5 µM and **18**–**22**: 50 µM). The thermal melting points (*T_m_*, °C) were determined as the maxima of the first derivative plots of the melting curves.

#### Fluorescence spectroscopy

Fluorescence spectra of the studied 7-MEOTA-THA thio-/ureas were recorded in a quartz cuvette (1 cm path length) on a Varian Cary Eclipse spectrofluorimeter. The widths of both the excitation and emission slit were set at 10 nm. The excitation wavelengths were 340 nm for compounds **13**–**21** and 335 nm for compounds **12** and **22**. The emission spectra were recorded in the range 350–550 nm with maximum observed at 376–396 nm. Fluorescence titrations were realized in 10 mM Tris–HCl buffer (pH 7.4; RT) containing a constant concentration of the studied derivative (**12**–**22**: 5 µM), and to which were added equivalent increased amounts of ctDNA (0–280 µM). The steady state fluorescence measurements were performed at three different temperatures (288, 298 and 310 K) for the evaluation of various thermodynamic parameters important in the detection of complex formation between DNA and 7-MEOTA-THA heterodimers.

The obtained data were used for determination of Stern–Volmer quenching constants (***K_SV_***) by applying the equation (Equation (4)^73^:

(4) $$\frac{F_{0}}{F}=1+K_{\mathrm{SV}}\left[Q\right]$$

where F_0_ is the fluorescence intensity of the studied compound alone, F is the fluorescence intensity of the studied 7-MEOTA-THA thio-/urea in the presence of ctDNA as a quencher and [Q] is the molar concentration of ctDNA. To further confirm the quenching process were calculated biomolecular quenching constants k_q_ using the equation (Equation (5)):

(5) $$k_{q}=\frac{K_{\mathrm{SV}}}{\tau_{0}}$$

where τ_0_ is the fluorescence lifetime of the biomolecule in the absence of quencher, which is around 10^−8^ s^93^.

The modified Stern–Volmer equation (Equation (6)^94^ was used to determine binding constants (K_b_) of the forming complex between 7-MEOTA-THA thio-/urea hybrids and ctDNA:

(6) $$\log\frac{F_{0}-F}{F}=\log K_{b}+\text{n log}\left[Q\right]$$

where n is the number of binding sites. K_b_ and n were calculated at three different temperatures from double logarithm regression curves of log(F_0_−F)/F versus log[Q] and they were obtained from intercept and slope, respectively.

The van’t Hoff equation (Equation (7)) was used to calculate thermodynamic parameters, namely change of enthalpy (ΔH), change of entropy (ΔS) and change of free energy (ΔG)^95^:

(7) $$\log K_{b}=-\frac{\Delta H}{2.303\text{ RT}}+\frac{\Delta S}{2.303\;R}$$

where T is the temperature in kelvin and R is the universal gas constant equal to 8.314 kJ K^−1^ mol^−1^. ΔH and ΔS were determined as the slope and intercept respectively of the van’t Hoff plot (log K_b_ against 1/T). The ΔG values at three different concentrations were calculated using equation (Equation (8))^96^:

(8) ΔG=ΔH−TΔS.

#### Circular and LD spectroscopy

Spectral measurements of CD were realized using a J-810 Jasco spectropolarimeter in a quartz cuvette with optic length 1 mm. CD spectra of ctDNA (7.46 µM) in the absence or in the presence of the newly developed 7-MEOTA-THA thio-/urea hybrids (**12**–**22**: 0.3 mM) were recorded in 10 mM Tris–HCl buffer (pH 7.4; RT) in the wavelength range of 200–500 nm. The results are presented as the mean of at least three repeated measurements and obtained data were transferred to Grafit 7.0 (Erithacus Software, West Sussex, UK) for analysis.

Flow LD spectra were measured using a Jasco J-720 spectropolarimeter in a flow Couette cell adapted for LD measurements. The flow cell includes a fixed outer cylinder and a solid rotating quartz inner cylinder. These two cylinders are separated with a 0.5 mm gap (total path length was 1 mm). LD spectra of ctDNA (310 µM) in the absence or in the presence of the newly synthesized 7-MEOTA-THA thio-/urea derivatives (**12**–**22**: 0–0.1 mM) were recorded in 10 mM Tris–HCl buffer (pH 7.4; RT) in the wavelength range of 200–500 nm. The baseline (10 mM Tris–HCl) was also recorded for each measurement. The results are presented as the mean of at least three independent measurements and obtained data were transferred to Grafit 7.0 (Erithacus Software) for analysis.

### Chemistry

7-MEOTA was prepared at the University of Defense (Faculty of Military Health Sciences, Hradec Kralove, Czech Republic) by the previously described method^39^^,^^97^^,^^98^. Other reagents were obtained from Sigma-Aldrich (Prague, Czech Republic) in reagent grade quality. All experiments were carried out under nitrogen atmosphere. Thin-layer chromatography (TLC) was performed on aluminum sheets with pre-coated silica gel 60 F_254_ (Merck, Czech Republic). Column chromatography was performed at normal pressure on silica gel 100 (particle size 0.063–0.200 mm, 70–230 mesh ASTM; Fluka, Bucharest, Romania). Elemental analysis was measured on a Perkin-Elmer CHN Analyzer 2400 Series II apparatus (PerkinElmer, Waltham, MA). Mass spectra were recorded using combined high-performance liquid chromatography (HPLC) and mass spectrometry (MS). The HP1100 HPLC system was obtained from Agilent Technologies (Waldbronn, Germany). It consisted of vacuum degasser G1322A, quaternary pump G1311A, autosampler G1313A and quadrupole mass spectrometer MSD1456 VL equipped with electrospray ionization source. Nitrogen for MS was supplied by a Whatman 75–720 nitrogen generator. Data were collected in positive ion mode with an ESI probe voltage of 4000 V. The pressure of nebulizer gas was set to 35 psig. Drying gas temperature was operated at 335 °C and flow at 13 L/min. ^1^H NMR and ^13^C NMR spectra were recorded on a Varian Mercury Plus 400 spectrometer operating at 400 and 100 MHz, respectively, in deuterochloroform [CDCl_3_; 7.27 (D), 77.2 (C) ppm] or hexadeuterodimethylsulfoxide [DMSO-*d*_6_; 2.50 (D), 39.7 (C) ppm] using tetramethylsilane (TMS) as internal reference (=0 ppm for both nuclei). Chemical shifts are reported in parts per million (ppm, δ) relative to TMS. The assignment of chemical shifts is based on standard NMR experiments (^1^H, ^13^C, ^1^H−^1^H COSY, ^1^H−^13^C HSQC, HMBC, DEPT). Uncalibrated purity was ascertained by LC − UV using a reverse phase C18 chromatographic column. All of the biologically tested compounds exhibited purity 96 − 99% at wavelength 254 nm. Melting points were measured on a micro heating stage PHMK 05 (VEB Kombinat Nagema, Germany) and were uncorrected.

#### General procedure for synthesis of novel 7-MEOTA-THA heterodimers 12–23

Intermediates **3**–**8** (1.7 mmol) and **11** (0.5 g, 2.1 mmol) were added to 20 ml of dichloromethane and stirred for 48 h at room temperature (RT). The mixture was concentrated under reduced pressure to give the crude product. Purification was performed by flash chromatography (eluent CHCl_3_/MeOH, 9:1) to give **12**–**17** as a yellow solid. In a further step, the appropriate 7-MEOTA-THA product containing the thiourea moiety (**12**–**17**, 1.1 mmol) was stirred in dry dichloromethane (25 ml) with 2,4,6-trimethylbenzonitrile *N*-oxide (0.2 g, 1.2 mmol) for 48 h. The resulting mixture was subsequently evaporated under reduced pressure and purified by flash chromatography using CHCl_3_/MeOH (9:1) as eluent to yield **18**–**23** as white solids.

*1-(2-(7-Methoxy-1,2,3,4-tetrahydroacridin-9ylamino)ethyl)-3-(1,2,3,4-tetrahydroacridin-9-yl)thiourea****12***. Yellow solid (0.61 g, 69%): m.p.=142.1–143.2 °C; ^1^H NMR (CDCl_3_) δ 1.83 (m, 8H, 4 × CH_2_, H-2,3,2′′,3′′), 2.63 and 2.88 (m, 4H, 2 × CH_2_, H-1,1′′), 2.95 and 3.05 (m, 4H, 2 × CH_2_, H-4,4′′), 3.87 (s, 3H, OCH3), 3.60 and 3.66 (m, 4H, 2 × CH_2_, H-1′,2′), 7.33 (t, 1H, CH, H-7′′, *J* = 7.6 Hz), 7.20 (dd, 1H, CH, H-6, *J* = 9.2, 2.4 Hz), 7.27 (d, 1H, CH, H-8, *J* = 2.8 Hz), 7.56 (d, 1H, CH, H-6′′, *J* = 7.6 Hz), 7.74 (m, 1H, CH, H-8′′), 7.82 (d, 1H, CH, H-5, *J* = 9.2 Hz), 7.90 (d, 1H, CH, H-5′′, *J* = 8.8 Hz), 6.80 (bs, 1H, NH); ^13^C NMR (CDCl_3_) δ 21.8, 22.0, 22.2, 22.4 (C-2,3,2′′,3′′), 24.6 and 25.3 (C-1,1′′), 32.2 and 33.6 (C-4,4′′), 42.5 and 46.8 (C-1′,2′), 55.7 (OCH3), 101.9 (C-8), 116.2 (C-9a), 119.6 (C-8a), 121.6 (C-6), 122.6 (C-8′′), 124.2 (C-8a′′), 126.5 (C-7′′), 128.8 (C-5,5′′), 129.1 (C-9a′′), 129.3 (C-6′′), 139.8 (C-9′′), 143.2 (C-10a), 147.2 (C-10a′′), 150.2 (C-9), 156.2 (C-7,4a), 160.5 (C-4a′′), 181.5 (C = S); ESI-MS: m/z 512.2 [M]^+^ (calculated for: [C30H34N5OS]^+^ 512.2); Anal. Calcd. for C30H33N5OS: C, 70.42; H, 6.50; N, 13.69; S, 6.27. Found: C, 70.37; H, 6.81; N, 13.78; S, 6.05.

*1-(3-(7-Methoxy-1,2,3,4-tetrahydroacridin-9-ylamino)propyl)-3-(1,2,3,4-tetrahydroacridin-9-yl)thiourea****13***. Yellow solid (0.71 g, 78%): m.p.=106.3–107.5 °C; ^1^H NMR (CDCl_3_) δ 1.81 (m, 10H, 5 × CH_2_, H-2,3,3′,2′′,3′′), 2.50 and 2.70 (m, 4H, 2 × CH_2_, H-1), 2.94 (m, 4H, 2 × CH_2_, H-4,4′′), 3.24 and 3.48 (m, 4H, 2 × CH_2_, H-1′,3′), 3.85 (m, 3H, OCH_3_), 5.65 (bs, 1H, NH), 7.17 (m, 2H, 2 × CH, H-6,8), 7.33 (m, 1H, CH, H-7′′), 7.51 (m, 1H, CH, H-6′′), 7.81 (m, 1H, CH, H-8′′), 7.83 (m, 1H, CH, H-5), 7.94 (m, 1H, CH, H-5′′); ^13^C NMR (CDCl_3_) δ 22.0, 22.2, 22.4, 22.6 (C-2,3,2′′,3′′), 24.8 and 25.3 (C-1,1′′), 30.6 (C-2′), 33.6 (C-4,4′′), 43.3 and 42.3 (C-1′,3′), 55.7 (OCH3), 101.5 (C-8), 116.4 (C-9a), 120.6 (C-8a), 121.6 (C-6), 122.6 (C-8′′), 124.4 (C-8a′′), 126.3 (C-7′′), 128.5 (C-5,5′′), 129.1 (C-6′′,9a′′), 139.0 (C-9′′), 143.0 (C-10a), 147.0 (C-10a′′), 150.5 (C-9), 156.3 (C-7,4a), 160.3 (C-4a′′), 181.5 (C = S); ESI-MS: *m/z* 526.2 [M]^+^ (calculated for: [C31H36N5OS]^+^ 526.3); Anal. Calcd. for C31H35N5OS: C, 70.82; H, 6.71; N, 13.32; S, 6.10. Found: C, 70.65; H, 6.67; N, 13.45; S, 6.01.

*1-(4-(7-Methoxy-1,2,3,4-tetrahydroacridin-9-ylamino)butyl)-3-(1,2,3,4-tetrahydroacridin-9-yl)thiourea****14***. Yellow solid (0.54 g, 58%): m.p.=95.6–96.4 °C; ^1^H NMR (CDCl_3_) δ 1.64 (m, 4H, 2 × CH_2_, H-2′,3′), 1.85 (m, 8H, 4 × CH_2_, H-2,3,2′′,3′′), 2.64 and 2.87 (m, 4H, 2 × CH_2_, H-1,1′′), 2.98 and 3.04 (m, 4H, 2 × CH_2_, H-4, 4′′), 3.43 and 3.64 (m, 4H, 2 × CH_2_, H-1′,4′), 3.87 (s, 3H, OCH_3_), 5.46 (bs, 1H, NH), 7.17 (d, 1H, CH, H-8, *J* = 2.8 Hz), 7.20 (dd, 1H, CH, H-6, *J* = 8.8, 2.8 Hz), 7.40 (t, 1H, CH, H-7′′, *J* = 7.6 Hz), 7.60 (t, 1H, CH, H-6′′, *J* = 7.6 Hz), 7.81 (m, 1H, CH, H-8′′), 7.83 (m, 1H, CH, H-5), 7.94 (d, 1H, CH, H-5′′, *J* = 8.8 Hz); ^13^C NMR (CDCl3) δ 22.2, 22.4, 22.6, 22.8 (C-2,3,2′′,3′′), 24.7 and 25.3 (C-1,1′′), 26.5 and 28.6 (C-2′,3′), 32.8 and 33.8 (C-4, 4′′), 44.8 and 48.1 (C-1′,4′), 55.6 (OCH3), 101.7 (C-8), 117.0 (C-9a), 120.7 (C-8a), 120.9 (C-6), 122.5 (C-8′′), 124.1 (C-8a′′), 126.7 (C-7′′), 128.8 (C-5,5′′), 129.1 (C-9a′′), 129.4 (C-6′′), 139.0 (C-9′′), 142.5 (C-10a), 147.3 (C-10a′′), 150.3 (C-9), 156.2 (C-7,4a), 160.6 (C-4a′′), 181.4 (C=S); ESI-MS: *m/z* 540.2 [M]^+^ (calculated for: [C32H38N5OS]^+^ 540.3); Anal. Calcd. for C32H37N5OS: C, 71.21; H, 6.91; N, 12.98; S, 5.94. Found: C, 71.19; H, 6.55; N, 13.26; S, 5.80.

*1-(5-(7-Methoxy-1,2,3,4-tetrahydroacridin-9-ylamino)pentyl)-3-(1,2,3,4-tetrahydroacridin-9-yl)thiourea****15***. Yellow solid (0.78 g, 81%): m.p.=93.2–94.1 °C; ^1^H NMR (CDCl_3_) δ 1.43 (m, 2H, CH_2_, H-3′), 1.59 and 1.71 (m, 4H, 2 × CH_2_, H-2′,4′), 1.83 (m, 8H, 4 × CH_2_, H-2,3,2′′,3′′), 2.63 (m, 2H, CH_2_, H-1), 2.95 (m, 6H, 3 × CH_2_, H-4,1′′,4′′), 3.57 (m, 4H, 2 × CH_2_, H-1′,5′), 3.87 (s, 3H, OCH3), 7.18 (dd, 1H, CH, H-6, *J* = 9.2, 2.4 Hz), 7.27 (m, 1H, CH, H-8), 7.33 (m, 1H, CH, H-7′′), 7.54 (m, 1H, CH, H-6′′), 7.82 (m, 2H, 2 × CH, H-5,8′′), 7.90 (d, 1H, CH, H-5′′, *J* = 8.4 Hz); 13C NMR (CDCl_3_) δ 21.8, 22.2, 22.5, (C-2,3,2′′,3′′), 24.0 (C-3′), 24.6 and 25.3 (C-1,1′′), 28.6 and 30.9 (C-2′,4′), 31.4 and 33.8 (C-4,4′′), 44.8 and 48.3 (C-1′,5′), 55.7 (OCH_3_), 102.3 (C-8), 117.0 (C-9a), 119.6 (C-8a), 121.6 (C-6), 122.6 (C-8′′), 124.3 (C-8a′′), 126.4 (C-7′′), 128.6 and 128.7 (C-5,5′′), 129.2 (C-6′′,9a′′), 139.8 (C-9′′), 143.5 (C-10a), 147.1 (C-10a′′), 151.8 (C-9), 156.3 (C-7,4a), 160.4 (C-4a′′), 181.5 (C = S); ESI-MS: *m/z* 554.3 [M]^+^ (calculated for: [C33H40N5OS]^+^ 554.3); Anal. Calcd. for C33H39N5OS: C, 71.57; H, 7.10; N, 12.65; S, 5.79. Found: C, 71.23; H, 6.95; N, 12.88; S, 5.93.

*1-(6-(7-Methoxy-1,2,3,4-tetrahydroacridin-9-ylamino)hexyl)-3-(1,2,3,4-tetrahydroacridin-9-yl)thiourea****16***. Yellow solid (0.92 g, 93%): m.p.=74.2–74.8 °C; ^1^H NMR (CDCl_3_) δ 1.25 and 1.36 (m, 4H, 2 × CH_2_, H-3′,4′), 1.50 and 1.61 (m, 4H, 2 × CH_2_, H-2′,5′), 1.89 (m, 8H, 4 × CH_2_, H-2,3,2′′,3′′), 2.65 and 2.88 (m, 4H, 2 × CH_2_, H-1,1′′), 2.97 and 3.07 (m, 4H, 2 × CH2, H-4,4′′), 3.44 and 3.58 (m, 4H, 2 × CH_2_, H-1′,6′), 3.88 (s, 3H, OCH_3_), 7.20 (m, 1H, CH, H-6), 7.27 (m, 1H, CH, H-8), 7.41 (dd, 1H, CH, H-7′′, *J* = 8.4, 6.8 Hz), 7.59 (dd, 1H, CH, H-6′′, *J* = 8.4, 6.8 Hz), 7.82 (m, 2H, 2 × CH, H-5,8′′), 7.94 (d, 1H, CH, H-5′′, *J* = 8.4 Hz); ^13^C NMR (CDCl_3_) δ 22.2, 22.3, 22.5, 22.7 (C-2,3,2′′,3′′), 24.6 and 25.3 (C-1,1′′), 26.3 and 26.4 (C-3′,4′), 28.8 and 31.4 (C-2′,5′), 32.6 and 33.8 (C-4,4′′), 45.1 and 48.6 (C-1′,6′), 55.5 (OCH_3_), 102.0 (C-8), 116.1 (C-9a), 120.4 (C-8a), 120.8 (C-6), 122.5 (C-8′′),124.2 (C-8a′′), 126.5 (C-7′′), 128.3 (C-5), 128.7 (C-5′′), 129.0 (C-9a′′), 129.3 (C-6′′), 138.2 (C-9′′), 142.0 (C-10a), 147.3 (C-10a′′), 150.7 (C-9), 156.0 (C-7), 154.8 (C-4a), 160.5 (C-4a′′), 181.2 (C = S); ESI-MS: *m/z* 568.3 [M]^+^ (calculated for: [C34H42N5OS]^+^ 554.3); Anal. Calcd. for C33H39N5OS: C, 71.57; H, 7.10; N, 12.65; S, 5.79. Found: C, 71.23; H, 6.95; N, 12.88; S, 5.93.

*1-(7-(7-Methoxy-1,2,3,4-tetrahydroacridin-9-ylamino)heptyl)-3-(1,2,3,4-tetrahydroacridin-9-yl)thiourea****17***. Yellow solid (0.86 g, 85%): m.p.=80.8–81.9 °C; ^1^H NMR (CDCl_3_) δ 1.32 (m, 6H, 3 × CH_2_, H-3′,4′,5′), 1.43 and 1.63 (m, 4H, 2 × CH_2_, H-2′,6′), 1.85 (m, 8H, 4 × CH_2_, H-2,3,2′′,3′′), 2.64 and 2.90 (m, 4H, 2 × CH_2_, H-1,1′′), 2.97 and 3.10 (m, 4H, 2 × CH_2_, H-4,4′′), 3.52 (m, 4H, 2 × CH_2_, H-1′,7′), 3.88 (s, 3H, OCH_3_), 7.20 (dd, 1H, CH, H-6, *J* = 8.8, 2.0 Hz), 7.27 (m, 1H, CH, H-8), 7.40 (m, 1H, CH, H-7′′), 7.58 (t, 1H, CH, H-6′′, *J* = 7.6 Hz), 7.84 (m, 2H, 2 × CH, H-5,8′′), 7.94 (d, 1H, CH, H-5′′, *J* = 8.4 Hz); ^13^C NMR (CDCl_3_) δ 22.0, 22.2, 22.3, 22.6 (C-2,3,2′′,3′′), 24.5 and 25.3 (C-1,1′′), 26.5 (C-4′), 28.6 and 28.7 (C-3′,5′), 30.3 and 31.3 (C-2′,6′), 31.7 and 33.9 (C-4,4′′), 45.2 and 48.6 (C-1′,7′), 55.6 (OCH3), 102.3 (C-8), 115.5 (C-9a), 120.0 (C-8a), 121.4 (C-6), 122.6 (C-8′′), 124.8 (C-8a′′), 126.4 (C-7′′), 128.7 (C-5,5′′), 129.2 (C-6′′,9a′′), 139.7 (C-9′′), 145.2 (C-10a), 147.3 (C-10a′′), 151.3 (C-9), 156.2 (C-7,4a), 160.5 (C-4a′′), 181.3 (C = S); ESI-MS: *m/z* 582.3 [M]^+^ (calculated for: [C35H44N5OS]^+^ 582.3); Anal. Calcd. for C35H43N5OS: C, 72.25; H, 7.45; N, 12.04; S, 5.51. Found: C, 72.43; H, 7.58; N, 12.12; S, 5.43.

*1-(2-(7-Methoxy-1,2,3,4-tetrahydroacridin-9-ylamino)ethyl)-3-(1,2,3,4-tetrahydroacridin-9-yl)urea****18***. White solid (0.53 g, 95%): m.p.=103.7–104.8 °C; ^1^H NMR (CDCl_3_) δ1.70 (m, 8H, 4 × CH_2_, H-2,3,2′′,3′′), 2.66 and 2.81 (m, 4H, 2 × CH_2_, H-1,1′′), 3.0 (m, 4H, 2 × CH2, H-4,4′′), 3.66 and 3.93 (m, 4H, 2 × CH_2_, H-1′,2′), 3.85 (s, 3H, OCH_3_), 7.20 (m, 1H, CH, H-6), 7.26 (m, 1H, CH, H-7′′), 7.31 (m, 1H, CH, H-8), 7.42 (t, 1H, CH, H-6′′, *J* = 7.6 Hz), 7.83 (m, 2H, 2 × CH, H-5′′,8′′), 7.91 (bs, 1H, NH), 8.0 (d, 1H, CH, H-5, *J* = 8.8 Hz), 9.05 (bs, 1H, NH); ^13^C NMR (CDCl_3_) δ 22.2, 22.4, 22.5, 22.6 (C-2,3,2′′,3′′), 25.4 (C-1,1′′), 28.4 and 33.8 (C-4,4′′), 40.1 and 51.5 (C-1′,2′), 55.2 (OCH_3_), 102.1 (C-8), 116.5 (C-9a), 119.8 (C-8a), 121.1 (C-6), 123.2 (C-8′′), 125.3 (C-7′′), 124.5 (C-8a′′), 127.2 (C-5), 128.0 (C-5′′), 128.3 (C-6′′), 128.7 (C-9a′′), 139.9 (C-9′′), 146.6 (C-10a,10a′′), 150.0 (C-9), 155.4 (C-4a), 156.3 (C-7), 157.1 (C=O), 159.5 (C-4a′′); ESI-MS: *m/z* 496.2 [M]^+^ (calculated for: [C30H34N5O2]^+^ 496.3); Anal. Calcd. for C30H33N5O2: C, 72.70; H, 6.71; N, 14.13. Found: C, 72.43; H, 6.35; N, 13.69.

*1-(3-(7-Methoxy-1,2,3,4-tetrahydroacridin-9-ylamino)propyl)-3-(1,2,3,4-tetrahydroacridin-9-yl)urea****19***. White solid (0.52 g, 91%): m.p.=104.5–105.4 °C; ^1^H NMR (CDCl_3_) δ 1.72 (m, 10H, 5 × CH_2_, H-2,3,2′,2′′,3′′), 2.56 and 2.77 (m, 4H, 2 × CH_2_, H-1,1′′), 2.84 and 2.95 (m, 4H, 2 × CH_2_, H-4,4′′), 3.34 and 3.50 (m, 4H, 2 × CH_2_, H-1′,3′), 3.85 (m, 3H, OCH_3_), 6.01 (bs, 1H, NH), 7.20 (dd, 1H, CH, H-6, *J* = 8.8, 2.4 Hz), 7.26 (t, 1H, CH, H-7′′, *J* = 7.2 Hz), 7.30 (m, 1H, CH, H-8), 7.43 (dd, 1H, CH, H-6′′, *J* = 8.4, 7.2 Hz), 7.70 (d, 1H, CH, H-5, *J* = 8.8 Hz), 7.82 (m, 2H, 2 × CH, H-5′′,8′′), 8.35 (bs, 1H, NH); ^13^C NMR (CDCl_3_) δ 22.4, 22.6 (C-2,3,2′′,3′′), 25.3 (C-1,1′′), 31.2 (C-2′), 32.1 and 33.8 (C-4,4′′), 36.7 and 44.0 (C-1′,3′), 55.4 (OCH_3_), 101.5 (C-8), 114.6 (C-9a), 119.9 (C-8a), 122.0 (C-6), 122.9 (C-8′′), 124.6 (C-8a′′), 125.3 (C-7′′), 127.3 (C-5), 128.2 (C-5′′), 128.4 (C-6′′,9a′′), 139.8 (C-9′′), 146.8 (C-10a,10a′′), 152.1 (C-9), 153.3 (C-4a), 156.1 (C-7), 157.6 (C=O), 159.7 (C-4a′′); ESI-MS: *m/z* 510.2 [M]^+^ (calculated for: [C31H36N5O2]^+^ 510.3); Anal. Calcd. for C31H35N5O2: C, 73.06; H, 6.92; N, 13.74. Found: C, 73.42; H, 6.86; N, 13.95.

*1-(4-(7-Methoxy-1,2,3,4-tetrahydroacridin-9-ylamino)butyl)-3-(1,2,3,4-tetrahydroacridin-9-yl)urea****20***. White solid (0.56 g, 94%): m.p.=69.2–69.8 °C; ^1^H NMR (CDCl_3_) δ 1.70 (m, 4H, 2 × CH_2_, H-2′,3′), 1.80 (m, 8H, 4 × CH_2_, H-2,3,2′′,3′′), 2.57 and 2.80 (m, 4H, 2 × CH_2_, H-1,1′′), 2.90 and 3.0 (m, 4H, 2 × CH_2_, H-4,4′′), 3.40 and 3.50 (m, 4H, 2 × CH_2_, H-1′,4′), 3.85 (m, 3H, OCH_3_), 4.50 (bs, 1H, NH), 6.15 (bs, 1H, NH), 7.20 (dd, 1H, CH, H-6, *J* = 8.8, 2.4 Hz), 7.26 (t, 1H, CH, H-7′′, *J* = 7.2 Hz), 7.30 (m, 1H, CH, H-8), 7.43 (dd, 1H, CH, H-6′′, *J* = 8.4, 7.2 Hz), 7.75 (d, 1H, CH, H-5, *J* = 8.8 Hz), 7.84 (m, 2H, 2 × CH, H-5′′,8′′); ^13^C NMR (CDCl_3_) δ 22.1, 22.4, 22.5, 22.6 (C-2,3,2′′,3′′), 24.6 and 25.3 (C-1,1′′), 27.5 and 28.5 (C-2′,3′), 32.0 and 33.8 (C-4,4′′), 39.5 and 48.1 (C-1′,4′), 55.5 (OCH3), 102.1 (C-8), 115.6 (C-9a), 120.1 (C-8a), 121.1 (C-6), 122.7 (C-8′′), 124.4 (C-8a′′), 125.4 (C-7′′), 127.4 (C-9a′′), 128.2 (C-5), 128.5 (C-5′′,6′′), 139.5 (C-9′′), 146.8 (C-10a,10a′′), 151.1 (C-9), 154.2 (C-4a), 156.1 (C-7), 156.4 (C=O), 159.7 (C-4a′′); ESI-MS: *m/z* 524.3 [M]^+^ (calculated for: [C32H38N5O2]^+^ 524.3); Anal. Calcd for C32H37N5O2: C, 73.39; H, 7.12; N, 13.37. Found: C, 73.68; H, 7.34; N, 13.48.

*1-(5-(7-Methoxy-1,2,3,4-tetrahydroacridin-9-ylamino)pentyl)-3-(1,2,3,4-tetrahydroacridin-9-yl)urea****21***. White solid (0.58 g, 95%): m.p.=114.2–114.9 °C; ^1^H NMR (CDCl_3_) δ 1.43 (m, 2H, CH_2_, H-3′), 1.55 and 1.66 (m, 4H, 2 × CH_2_, H-2′,4′), 1.80 (m, 8H, 4 × CH_2_, H-2,3,2′′,3′′), 2.56 and 2.73 (m, 4H, 2 × CH_2_, H-1,1′′), 2.98 and 3.02 (m, 4H, 2 × CH_2_, H-4,4′′), 3.30 (m, 4H, 2 × CH_2_, H-1′,5′), 3.85 (s, 3H, OCH_3_), 5.62 (bs, 1H, NH), 7.20 (m, 1H, CH, H-6), 7.26 (m, 1H, CH, H-7′′), 7.27 (m, 1H, CH, H-8), 7.43 (m, 1H, CH, H-6′′), 7.67 (bs, 1H, NH), 7.77 (d, 1H, CH, H-5, *J* = 8.4 Hz), 7.82 (m, 1H, CH, H-8′′), 7.84 (d, 1H, CH, H-5′′, *J* = 8.8 Hz); ^13^C NMR (CDCl_3_) δ 22.9, 23.1 (C-2,3,2′′,3′′), 24.8 (C-3′), 24.7 and 25.4 (C-1,1′′), 29.0 and 33.4 (C-4,4′′), 30.7 and 31.2 (C-2′,4′), 40.4 and 48.5 (C-1′,5′), 56.4 (OCH_3_), 102.2 (C-8), 115.7 (C-9a), 120.3 (C-8a), 121.0 (C-6), 122.8 (C-8′′), 124.5 (C-8a′′), 125.3 (C-7′′), 127.3 (C-5), 127.8 (C-9a′′), 128.2 (C-5′′), 128.5 (C-6′′), 139.2 (C-9′′), 143.5 (C-10a), 145.8 (C-10a′′), 150.5 (C-9), 154.9 (C-4a), 156.1 (C-7), 156.3 (C=O), 160.1 (C-4a′′); ESI-MS: *m/z* 538.3 [M]^+^ (calculated for: [C33H40N5O2]^+^ 538.3); Anal. Calcd for C33H39N5O2: C, 73.71; H, 7.31; N, 13.02. Found: C, 73.39; H, 7.35; N, 13.33.

*1-(6-(7-Methoxy-1,2,3,4-tetrahydroacridin-9-ylamino)hexyl)-3-(1,2,3,4-tetrahydroacridin-9-yl)urea****22***. White solid (0.58 g, 93%): m.p.=91.4–92.1 °C; ^1^H NMR (CDCl_3_) δ 1.32 (m, 4H, 2 × CH_2_, H-3′,4′), 1.57 (m, 4H, 2 × CH_2_, H-2′,5′), 1.80 (m, 8H, 4 × CH_2_, H-2,3,2′′,3′′), 2.57 and 2.77 (m, 4H, 2 × CH_2_, H-1,1′′), 2.90 and 2.97 (m, 4H, 2 × CH_2_, H-4,4′′), 3.10 and 3.40 (m, 4H, 2 × CH_2_, H-1′,6′), 3.85 (s, 3H, OCH_3_), 4.52 (bs, 1H, NH), 5.82 (bs, 1H, NH), 7.15 (dd, 1H, CH, H-6, *J* = 9.2, 2.8 Hz), 7.23 (m, 2H, 2 × CH, H-8,7′′), 7.43 (dd, 1H, CH, H-6′′, *J* = 8.4, 7.2 Hz), 7.68 (bs, 1H, NH), 7.74 (d, 1H, CH, H-5, *J* = 9.2 Hz), 7.82 (m, 2H, 2 × CH, H-5′′,8′′); ^13^C-NMR (CDCl_3_) δ 22.1, 22.4, 22.7 (C-2,3,2′′,3′′), 24.6 and 25.3 (C-1,1′′), 26.2 (C-3′,4′), 29.9 and 31.3 (C-2′,5′), 32.1 and 33.8 (C-4,4′′), 39.8 and 48.2 (C-1′,6′), 55.6 (OCH_3_), 102.2 (C-8), 115.5 (C-9a), 120.1 (C-8a), 121.1 (C-6), 122.8 (C-8′′), 124.5 (C-8a′′), 125.4 (C-7′′), 127.4 (C-9a′′), 127.5 (C-5), 128.2 (C-5′′), 128.5 (C-6′′), 139.6 (C-9′′), 140.5 (C-10a), 146.8 (C-10a′′), 151.2 (C-9), 154.2 (C-4a), 156.1 (C-7), 156.4 (C=O), 159.7 (C-4a′′); ESI-MS: *m/z* 552.3 [M]^+^ (calculated for: [C34H42N5O2] + 552.3); Anal. Calcd for C34H41N5O2: C, 74.02; H, 7.49; N, 12.69. Found: C, 74.48; H, 7.15; N, 12.88.

*1-(7-(7-Methoxy-1,2,3,4-tetrahydroacridin-9-ylamino)heptyl)-3-(1,2,3,4-tetrahydroacridin-9-yl)urea****23***. White solid (0.61 g, 96%): m.p.=66.4–67.4 °C; ^1^H NMR (CDCl_3_) δ 1.25 (m, 6H, 3 × CH_2_, H-3′,4′,5′), 1.58 and 1.74 (m, 4H, 2 × CH_2_, H-2′,6′), 1.80 (m, 8H, 4 × CH_2_, H-2,3,2′′,3′′), 2.62 and 2.77 (m, 4H, 2 × CH_2_, H-1,1′′), 2.93 and 3.0 (m, 4H, 2 × CH_2_, H-4,4′′), 3.08 (m, 2H, CH_2_, H-7′), 3.41 (t, 2H, CH_2_, H-1′, *J* = 7.2 Hz), 3.86 (s, 3H, OCH_3_), 5.62 (bs, 1H, NH), 7.18 (dd, 1H, CH, H-6, *J* = 9.2, 2.4 Hz), 7.26 (m, 2H, 2 × CH, H-8,7′′), 7.46 (dd, 1H, CH, H-6′′, *J* = 6.8, 1.2 Hz), 7.57 (bs, 1H, NH), 7.77 (d, 2H, CH, H-5, *J* = 9.2 Hz), 7.83 (m, 2H, 2 × CH, H-5′′,8′′); ^13^C NMR (CDCl_3_) δ 22.3, 22.4, 22.6, 22.7 (C-2,3,2′′,3′′), 24.5 and 25.3 (C-1,1′′), 26.6 (C-4′), 28.6 and 28.8 (C-3′,5′), 30.3 and 31.3 (C-2′,6′), 32.4 and 33.8 (C-4,4′′), 40.0 and 48.6 (C-1′,7′), 55.5 (OCH_3_), 102.1 (C-8), 115.8 (C-9a), 120.3 (C-8a), 120.9 (C-6), 122.8 (C-8′′), 124.5 (C-8a′′), 125.4 (C-7′′), 127.4 (C-5), 128.1 (C-9a′′), 128.5 (C-6′′,5′′), 139.5 (C-9′′), 141.1 (C-10a), 146.8 (C-10a′′), 150.9 (C-9), 154.6 (C-4a), 156.0 (C-7), 156.3 (C=O), 159.7 (C-4a′′); ESI-MS: *m/z* 566.3 [M]^+^ (calculated for: [C35H44N5O2]^+^ 566.4); Anal. Calcd for C35H43N5O2: C, 74.30; H, 7.66; N, 12.38. Found: C, 74.55; H, 7.95; N, 12.36.

## Conclusion

In this study, we developed novel 7-MEOTA-THA heterodimers **12**–**22** combining the two fragments via a linker of varying length and containing either urea or thiourea moieties. In conclusion, extensive comparative biochemical, biophysical and biological measurements consistently indicated that **12**–**22** acted as DNA-binders and inhibitors of Topo I/II, and were able to affect the MMP and viability of HL-60 cells and suppress selectively cancer cell proliferation.

In summary, spectroscopic data proved that linking of THA and 7-MEOTA with an alkyl linker containing thio-/urea moieties positively influenced the DNA interaction power. From UV–vis absorption spectroscopic titration were calculated DNA-binding constants K = 0.5–8.0 × 10^6^ M^−1^. The obtained K for the heterodimers complexed with ctDNA were higher than K for THA (K = 3.8 × 10^4^ M^−1^) and 7-MEOTA (K = 7.9 × 10^4^ M^−1^) complexed with ctDNA, and moreover were comparable with K for the acridine–ctDNA complex (K = 4.5 × 10^6^ M^−1^). Data from fluorescence quenching experiments clearly demonstrated that ctDNA can quench fluorescence of the studied heterodimers, by static quenching. The latter correlates well with decreasing K_SV_ values and increasing temperatures and was further confirmed by obtained values of k_q_ (4.77–10.69 × 10^11^ M^−1^ s^−1^) which were higher than the limiting diffusion rate constant for dynamic quenching. Thermodynamic parameters (ΔH > 0, ΔS > 0) indicated that hydrophobic interactions play the key role in the binding of novel compounds to ctDNA, except in the case of heterodimers **16** and **20** which revealed a different pattern of negative ΔH and ΔS, pointing to van der Waals associations with ctDNA. The existence of LD signals also demonstrated that **12**–**22** interact with DNA and become oriented after attachment to DNA.

Based on all the obtained biological positive outcomes, 7-MEOTA-THA thio-/ureas can be considered as interesting and potential drug candidates as anticancer agents. This assertion is supported by the fact that they are able to affect cancer cell lines, whereas they remained unattached in healthy non-cancer fibroblasts at the tested concentration. The more effective heterodimers are those which contain longer alkyl chains. The higher antiproliferative effect of the more lipophilic agents containing either pentyl or hexyl chains could be ascribed to better cell membrane penetration. In this regard, thiourea hybrid **17** and urea hybrid **22** can be highlighted as the most potent. The studied heterodimers **14**–**17** and **21**, **22** were able to evoke MMP dissipation after 24 h treatment and caused cell death with an efficiency between 80 and 100%. In addition, thioureas **14**–**17** significantly altered cell cycle progression and induced block of the cell cycle in S phase, and ureas **21**, **22** demonstrated an increase of cells in G1 phase.

We believe that our study can contribute towards further development of novel, more potent and highly selective antiproliferative agents with intercalating properties.

## Supplementary Material

Supplemental Material
