# Supplementary material for: In vitro investigating of anticancer activity of new 7-MEOTA-tacrine heterodimers
Source: J Enzyme Inhib Med Chem. 2019 Apr 2;34(1):877–97. doi: 10.1080/14756366.2019.1593159 (PMC6450562; doi:10.1080/14756366.2019.1593159)
Supplement: Supplemental Material [file IENZ_A_1593159_SM3309.pdf]

Supplementary information

In vitro investigating of anticancer activity of new 7-MEOTA-tacrine heterodimers

Jana Janockova[1,2,†], Jan Korabecny[2,4,†], Jana Plsikova[1,3], Katerina Babkova[2,4], Eva Konkolova[1], Dana Kucerova[5], Jana Vargova[5], Jan Koval[5], Rastislav Jendzelovsky[5], Peter Fedorocko[5], Jana Kasparkova[6], Viktor Brabec[6], Jan Rosocha[3], Ondrej Soukup[2,4], Slavka Hamulakova[7], Kamil Kuca[2] and Maria Kozurkova[1,2,*]

[1]Department of Biochemistry, Institute of Chemistry, Faculty of Science, P. J. Šafárik University, Kosice, Slovak Republic; [2]Biomedical Research Center, University Hospital Hradec Kralove, Hradec Kralove, Czech Republic; [3]Associated Tissue Bank, Faculty of Medicine, P.J. Šafárik University, Slovak Republic; [4]Department of Toxicology and Military Pharmacy, Faculty of Military Health Sciences University of Defence, Hradec Kralove, Czech Republic; [5]Department of Cellular Biology, Institute of Biology and Ecology, Faculty of Science, P. J. Šafárik University, Kosice, Slovak Republic; [6]Department of Biophysics, Faculty of Science, Palacký University, Olomouc, Czech Republic; [7]Department of Organic Chemistry, Institute of Chemistry, Faculty of Science, P. J. Šafárik University, Slovak Republic

[†]these authors contributed equally

*corresponding author, e-mail: maria.kozurkova@upjs.sk, Tel.: +421556222124, address: Department of Biochemistry, Institute of Chemistry, Faculty of Science, P. J. Šafárik University, Moyzesova 11, 040 01 Kosice, Slovak Republic

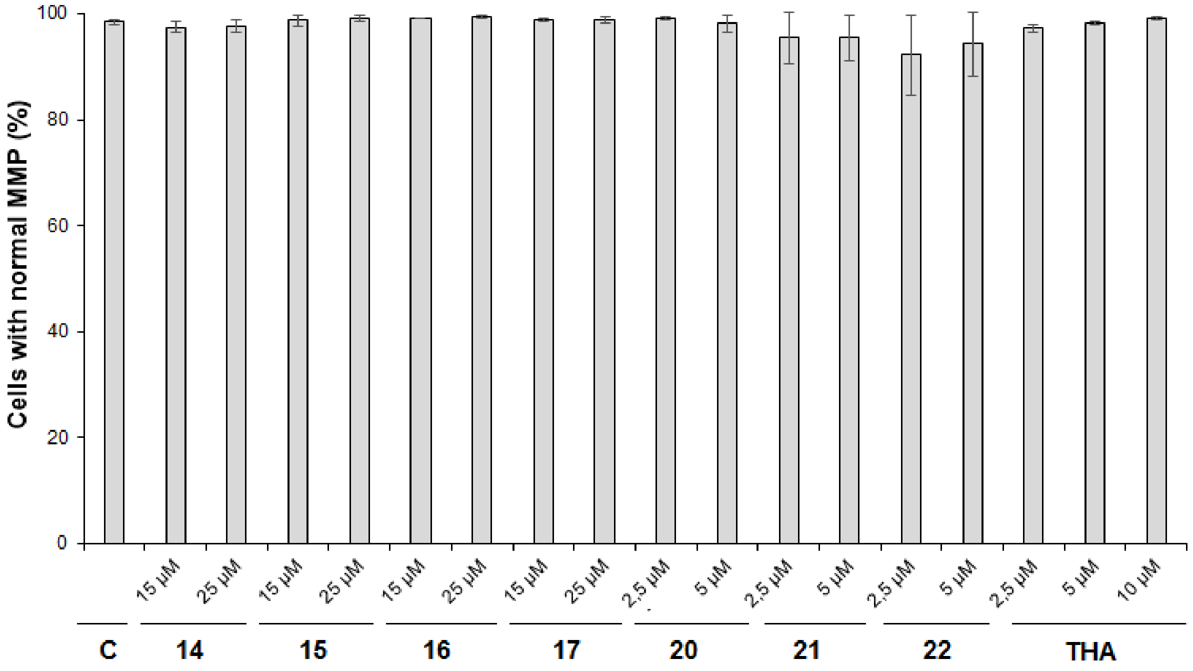

Figure S1. Effect of 7-MEOTA-THA thioureas **14** – **17** (15 – 25 µM), 7-MEOTA-THA ureas **20** – **22** (2.5 – 5 µM) and THA (2.5 – 10 µM) on changes in MMP of human dermal fibroblasts. Cells were analysed 72 h after treatment with studied compounds. The results were calculated as mean ± SD from three independent experiments. C = untreated control.

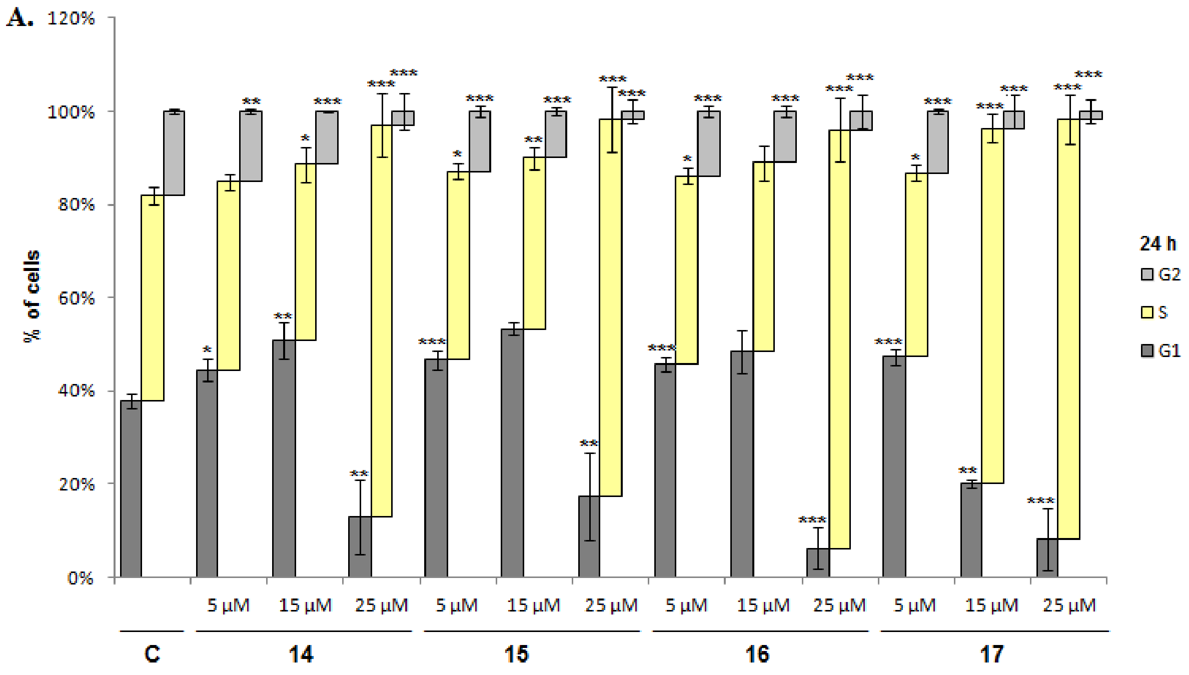

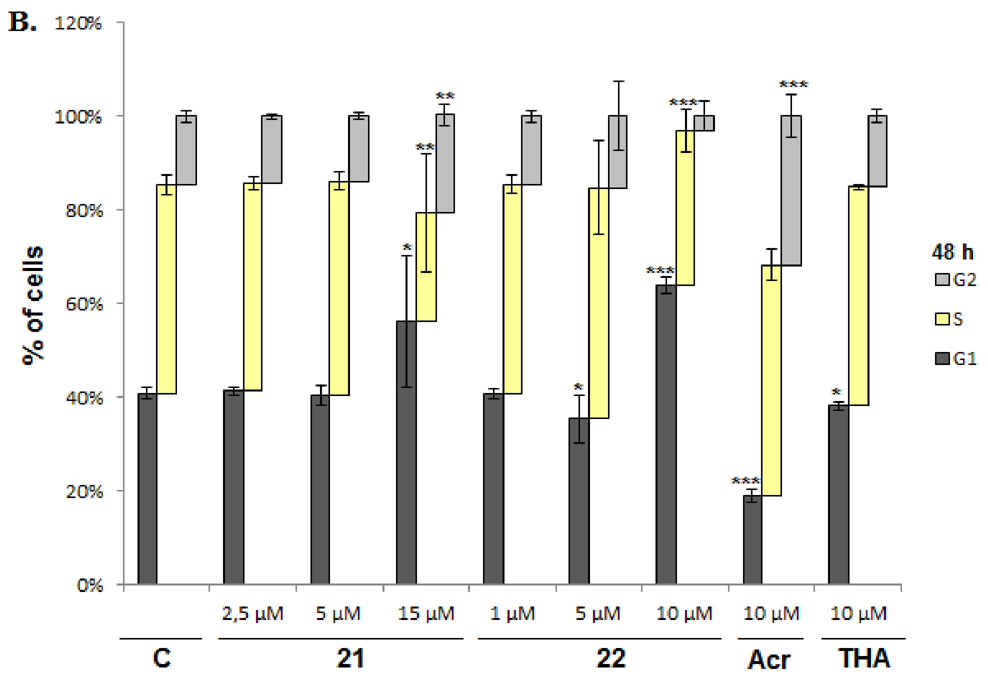

Figure S2. Effect of A: 7-MEOTA-THA thioureas **14** – **17** (5 – 25 µM), and B: 7-MEOTA-THA ureas **21** – **22** (1 – 15 µM) on cell cycle distribution in HL-60 cells. Cells were analyzed 24 h/ 48 h after treatment with studied compounds. DNA contents were analyzed using ModFit 3.0 software and results were calculated as mean ± SD from three independent experiments. Statistical significance $p < 0.05$ (*), 0.01 (**), 0.001 (***) for the particular experimental group compared to untreated control (C), Acr = acridine.

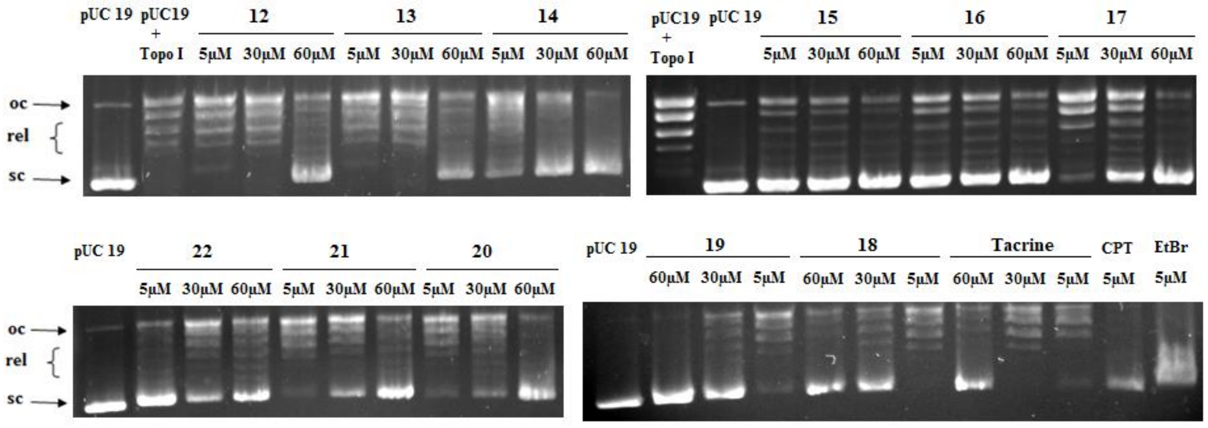

Figure S3. Effect of **12** – **22** on relaxation of supercoiled plasmid DNA by calf thymus Topo I. Supercoiled plasmid DNA pUC19 (lane pUC19) was incubated with calf thymus Topo I in the absence (lane pUC19 + Topo I) and in the presence of various compound concentrations (5, 30 and 60 µM) and CPT (=camptothecin) and EtBr (=ethidium bromide).

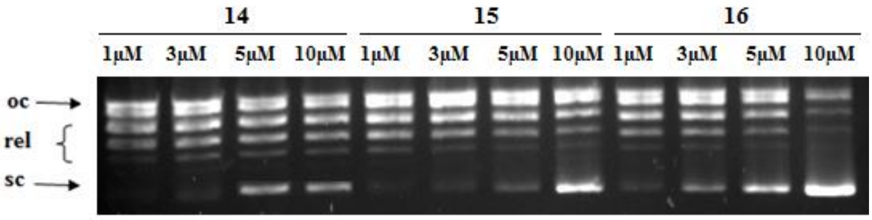

Figure S4. Effect of **14** – **17** on relaxation of supercoiled plasmid DNA by calf thymus Topo I. Supercoiled plasmid DNA pUC19 (lane pUC19) was incubated with calf thymus Topo I in the absence (lane pUC19 + Topo I) and in the presence of various compound concentrations (0 – 10 μM).

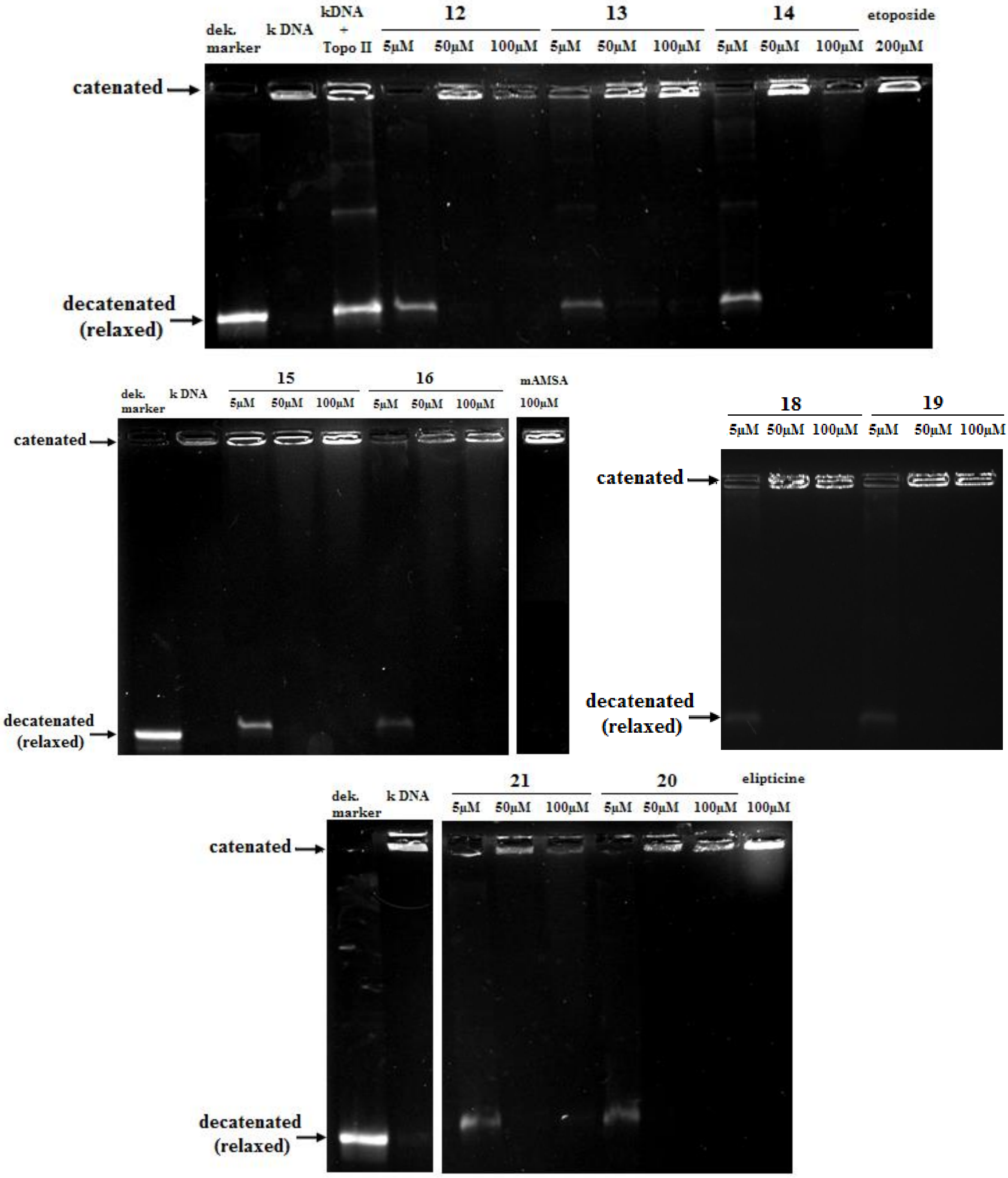

Figure S5. Effect of **12** – **21** (except for **17**) on decatenation of *k*DNA (0.16 µg) by human Topo IIα. Catenated kinetoplast DNA (lane *k*DNA) was incubated with human Topo IIα in the absence (lane *k*DNA + Topo II) and in the presence of various concentrations of compounds **12** – **21** (5 – 100 µM).

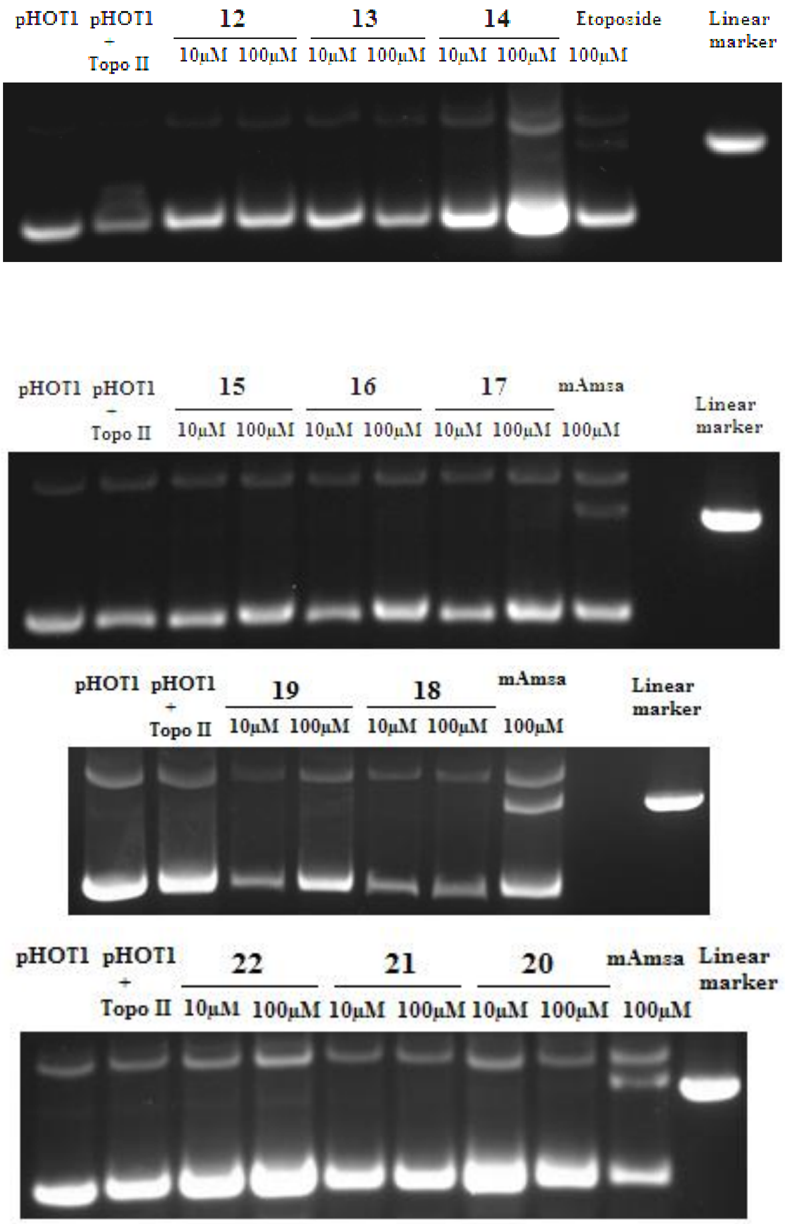

Figure S6. Effect of **12** – **22** on the accumulation of Topo II-DNA cleavage complexes. Plasmid DNA (lane pHOT1) was incubated with human Topo IIα (lane pHOT1 + Topo II) in the presence of compounds **12** – **22** (10 – 100 µM), etoposide (100 µM) or *m*AMSA (100 µM).

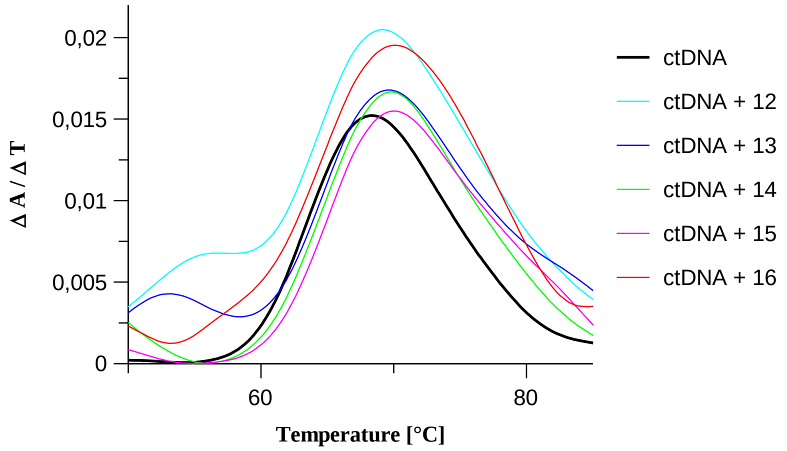

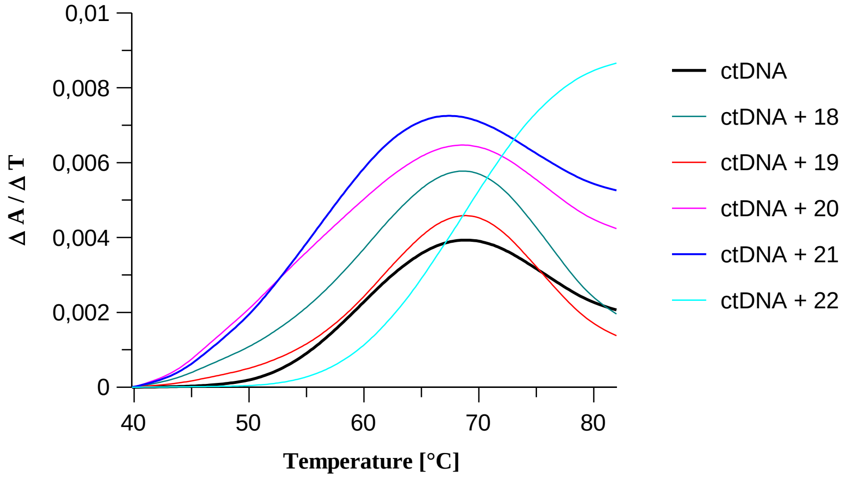

Figure S7. First derivative of ctDNA denaturation curve (black line, concentration of ctDNA was for thioureas 93.2 µM and for ureas 0.32 µM) in the presence of studied 7-MEOTA-THA thioureas **12** –**16** (colour lines, 37.5 µM) and ureas **18** – **22** (colour lines, 50 µM) which was measured at 260 nm in BPE buffer (pH 7.1).

Table S1. Channel designation for fluorescence microscopy with position of images from each channel. BP = bandpass filter, LP = long-pass filter.

**Channel designation/ image position**	**Filter cube**	**Excitation range**	**Excitation filter**	**Dichromatic mirror**	**Suppression filter**
0 (upper left)	Empty system	/	/	/	/
1 (upper right)	I3	Blue	BP 450-490	510	LP 515

Experimental

Materials

All chemicals and reagents purchased were of reagent grade and used without further purification.

Agarose (type II No-A-6877), ampicillin, glutathione (GSH, reduced form), ethylenediamine-tetraacetic acid (EDTA), ethidium bromide (EtBr), Hoechst 33342, 4-(2-hydroxyethyl)-1-piperazine-ethanesulfonic acid (HEPES), 3-(4,5-dimethylthiazolyl)-2,5-diphenyltetrazolium bromide (MTT), propidium iodide (PI), McCoy´s medium, tetramethylrhodamine ethyl ester perchlorate (TMRE), Triton X-100 and LB broth (microbial growth medium) were purchased from Sigma-Aldrich (Germany). Hefe Yeast extract, tris(hydroxymethyl)aminomethane (Tris) and tryptone - peptone were obtained from Roth (Germany). Amphotericin, penicillin and streptomycin were from Invitrogen (USA); fetal bovine serum (10 % FBS, heat activated) and RPMI medium from Gibco (USA); dimethyl sulfoxide (DMSO) and sodium dodecyl sulfate (SDS) were from Serva (Germany).

Calf thymus DNA (ctDNA) and plasmid pUC19 (2761 bp, bacterial strain DH 5α) were purchased from Sigma Aldrich (Germany). DNA Topo I from calf thymus and 10 × DNA Topo I buffer were obtained from Takara Bio Inc. (Japan). Catenated DNA (kDNA), kit for DNA Topo II, linear DNA, human DNA Topo II, plasmid pHOT-1, proteinase K, ribonuclease A and 10 × DNA Topo II buffer were supplied from TopoGen (USA).

Leukemic cell line HL-60 (ATCC #CCL-240, USA) was grown in suspension in a complete RPMI 1640 medium (Gibco, USA) supplemented with antibiotics (penicillin 100 U/mL, streptomycin 100 μg/mL and amphotericin 25 μg/mL; Invitrogen, USA) and 10% heat-inactivated fetal bovine serum (FBS, PAA Laboratories GmbH, Austria), maintained at 37 °C, 95% humidity and in a 5% CO2 atmosphere. Adherent adenocarcinoma cell line A549 (human lung alveolar basal epithelial cell line, ATCC, #CCL-185, USA) was grown in

Kaighn's modification of F-12 Ham Nutrient Mixture (Sigma-Aldrich, Germany) supplemented with 10% heat-inactivated FBS (Biosera, France) and antibiotics (1% antibiotic-antimycotic 100× and gentamicin 50 µg/mL; Biosera, France) at 37 °C, 95% humidity and under 5% CO_2.

Human fibroblasts were isolated from skin biopsy during abdominal surgery and placed in the transport medium containing phosphate-buffered saline solution (PBS) (Gibco, USA) supplemented with 1% (v/v) antibiotic/antimycotic solution (penicillin 100 U/mL, streptomycin 100 µg/mL and amphotericin B 0.25 µg/mL; Gibco, USA). The experiments were performed with the approval of the University's ethical committee. Epidermis was dissected from the skin biopsy and the dermis was cut into 2–3 mm2 pieces, digested with 0.1% (v/v) bacterial collagenase type II (Gibco, USA) at 37 °C for 6 h, and then filtered through a 40 µm cell strainer (Falcon, BD Biosciences, California). The filtrate was centrifuged at 300 g for 7 min. The cells were cultured at 37 °C with 5% CO_2 in Dulbecco's modified Eagle's medium (DMEM) (Biochrom AG, Germany) containing 1% (v/v) antibiotic/ antimycotic solution and supplemented with 10% FBS (Gibco, USA).
